# Definition dependence of questionnaire-reported maternal temperature elevation in a retrospective registry cohort of women receiving neuraxial labor analgesia

**DOI:** 10.3389/fmed.2026.1960868

**Published:** 2026-10-01

**Authors:** Sumei Hu, Yihan Zheng, Shihong Huang

**Affiliations:** Department of Anesthesiology, Fujian Maternity and Child Health Hospital, College of Clinical Medicine for Obstetrics & Gynecology, Fujian Medical University, Fuzhou, China

**Keywords:** body temperature, epidural analgesia, labor analgesia, maternal fever, missing outcome data, retrospective cohort, selection bias

## Abstract

**Background:**

Questionnaire-reported maternal temperature elevation during neuraxial labor analgesia may vary depending on the outcome definition. We compared nested definitions and examined exploratory associations while addressing incomplete outcome linkage.

**Methods:**

This retrospective registry-based cohort comprised 3,409 labor-analgesia records from July 2022 through March 2023, with 1,658 (48.6%) having a linked questionnaire temperature outcome. The outcomes reported were a fever at or above 38.0 °C and any elevation in temperature. We used Firth penalized logistic regression for the sparse strict-fever endpoint, conventional logistic regression for the broader endpoint, multiple imputation with 50 imputed datasets for missing covariates, and inverse probability of outcome observation weighting (IPOW) for linkage sensitivity.

**Results:**

Strict fever was reported in 88/1,658 linked records [5.3%; 95% confidence interval (CI), 4.3%–6.5%], and any elevation was noted in 287/1,658 (17.3%; 95% CI, 15.6%–19.2%). IPOW-standardized estimates were 5.4% and 17.5%, respectively. Among 1,139 complete records, nulliparity had a large but imprecise association with strict fever [Firth-adjusted odds ratio (OR), 8.05; 95% CI, 2.51–40.76], whereas the dural puncture epidural estimate crossed the null (OR, 0.60; 95% CI, 0.35–1.02). For any elevation, age (OR per year, 1.08; 95% CI, 1.03–1.13), nulliparity (OR, 3.23; 95% CI, 1.86–5.63), and analgesia duration (OR per 2 h, 1.13; 95% CI, 1.06–1.20) were associated with higher odds; duration non-linearity remained exploratory (*p* = 0.002).

**Conclusion:**

Observed questionnaire-reported temperature frequency was definition-dependent. Incomplete outcome linkage, sparse strict-fever events, uncertain temperature timing, and differential laboratory testing limit inference; the findings are exploratory and require prospective validation with continuous timestamped temperature measurement.

## 1 Introduction

Intrapartum maternal fever commonly prompts evaluation of neonatal sepsis (1). Among term infants, an increase in maternal temperature has been associated with adverse neonatal outcomes (2). Earlier cohort data have similarly linked maternal fever to transient neonatal morbidity (3). These observations motivate reliable maternal temperature phenotyping; however, the analysis export available for the present study contained no validated neonatal outcome data.

Epidural analgesia has long been associated with increased maternal temperature during labor (4). Current evidence supports a multifactorial syndrome in which labor duration, altered thermoregulation, sterile inflammation, and infection may overlap with each other (5). Local-anesthetic modulation of inflammatory and cytokine pathways has been proposed, but the relative contributions of these mechanisms remain uncertain (6). Mechanistic cohort studies have implicated leukocyte caspase-1 activity in a subset of women with epidural-related maternal fever (7). Clinical observations have further shown that epidural-related fever can occur with non-infectious inflammation at term (8).

A 2020 editorial framed epidural-related maternal fever as a topic with unresolved issues (9). Contemporary reviews distinguish epidural-related maternal fever from intraamniotic infection while recognizing that bedside separation can be difficult (10). A recent narrative review highlighted the continuing uncertainty regarding pathophysiology, clinical significance, preventive strategies, and treatment (11). Higher maximum intrapartum temperatures have been associated with increased neonatal morbidity (12). Preventive trials have not identified a uniformly effective intervention, increasing the importance of reproducible outcome definitions and measurements (13).

We therefore compared a strict threshold with a broader, nested questionnaire-derived temperature category in one registry cohort. Our objectives were to quantify the observed outcome frequency among linked records, describe how estimates changed with the definition, and examine exploratory maternal and analgesia-related associations. We did not aim to develop a clinical prediction model or to evaluate neonatal outcomes.

## 2 Materials and methods

### 2.1 Study design, setting, and participants

This was a single-center retrospective registry-based cohort that was incompletely linked to a questionnaire-derived outcome. The registry covered neuraxial labor analgesia at Fujian Maternity and Child Health Hospital, Fuzhou, China, from 1 July 2022 through 31 March 2023. Reporting adhered to the Strengthening the Reporting of Observational Studies in Epidemiology (STROBE) statement (14).

The locked export contained all 3,409 labor-analgesia records available to the investigators for the study period. A screening log and any eligibility or exclusion rules applied before export were unavailable; therefore, pre-export registry capture could not be reconstructed. Records without a linked temperature outcome were retained for linkage-bias assessment but were never classified as normal temperature.

Upstream questionnaires were matched deterministically using cleaned maternal names, maternal age bands, and a questionnaire update date, occurring 0–14 calendar days after the registry date; an existing study identifier was not used for this match. Registry age was grouped as 20 years or younger, 21–25, 26–30, 31–35, 36–40, or older than 40 years and matched to the standardized questionnaire age band. When a questionnaire had multiple candidate registry records, the smallest non-negative date difference was selected, with the registry row order breaking ties. When multiple questionnaires matched one registry record, a unique-candidate match was preferred, followed by the smallest date difference and questionnaire row order. Names, age bands, and dates were required for candidate generation, and records with missing linkage fields could not be matched. The upstream algorithm and aggregate linkage provenance were re-audited against the locked export.

### 2.2 Questionnaire-derived temperature outcomes

The strict outcome was questionnaire-reported maternal fever at or above 38.0 °C. The broader outcome was any questionnaire-reported temperature elevation and included both elevations below 38.0 °C and fever at or above 38.0 °C.

The source questionnaire item asked, in Chinese, “Did you develop fever after labor analgesia?” (English translation). The retained response categories were normal temperature, elevation below 38.0, 38.0–38.5, 38.6–39.0, and above 39.0 °C. The upstream files retained a platform update timestamp, which was used for linkage but was not validated as the time of questionnaire completion, temperature measurement, or fever onset. Respondent identity, completion procedures, the lower boundary of the below-38.0 °C category, measurement device or anatomical site, continuous trace, and clinical measurement timestamps could not be determined. It was not possible to determine whether each response reflected a contemporaneously measured value or a later recollection. The outcome is therefore consistently described as questionnaire-reported temperature elevation, and recall and reporting variability are acknowledged.

### 2.3 Covariates, temporal interpretation, and neonatal outcome availability

The 10 covariates carried forward from the submitted association model were maternal age, body mass index (BMI), nulliparity, cervical dilation at analgesia initiation, membrane rupture, gestational diabetes, hypertensive disorder, dural puncture epidural (DPE), total analgesia duration, and pre-analgesia white blood cell (WBC) count. Calendar time was added during the peer review because technique use and outcome recording changed across the nine-month registry period. Associations of baseline and initiation variables with the temperature outcomes were evaluated using a cohort framework. Because the temperature-onset time was unavailable, the total duration and post-analgesia laboratory variables had uncertain temporal ordering and were interpreted only as descriptive or cross-sectional associations.

Table 1 groups the available descriptive data into maternal characteristics, recorded antenatal complications, status, and data at analgesia initiation, and subsequent clinical course. It reports all registry records and outcome-linked and outcome-unlinked subsets. Pre-analgesia hemoglobin and platelet counts were included for descriptive completeness, not as additional model covariates; pre-analgesia laboratory values were the nearest available measurements before analgesia. Gestational diabetes and hypertensive disorders are indicators derived from linked diagnosis lists, and the absence from a list does not independently verify the absence of disease. For Table 1, missing source parity and unknown or other membrane statuses were excluded from the respective available-value denominators. The previously specified models and model-based summaries retain the locked binary indicators, in which the source entries were coded as not positive. Gestational age was not summarized because mixed source formats and implausible values could not be adjudicated, and unrecorded complications were not inferred.

**Table 1:** Maternal characteristics, recorded antenatal complications, and collected clinical data by questionnaire-outcome linkage status.

Characteristic	All registry records (*n* = 3,409)	Outcome-linked (*n* = 1,658)	Outcome-unlinked (*n* = 1,751)
Maternal characteristics			
Maternal age, years	29.8 (4.0); *n* = 3,393	29.6 (4.0); *n* = 1,658	29.9 (3.9); *n* = 1,735
Body mass index, kg/m^2^	26.0 (3.1); *n* = 3,388	25.9 (3.1); *n* = 1,653	26.2 (3.1); *n* = 1,735
Nulliparity	2,553/3,399 (75.1%)	1,217/1,657 (73.4%)	1,336/1,742 (76.7%)
Recorded antenatal complications			
Recorded gestational diabetes	626/3,409 (18.4%)	307/1,658 (18.5%)	319/1,751 (18.2%)
Recorded hypertensive disorder	143/3,409 (4.2%)	66/1,658 (4.0%)	77/1,751 (4.4%)
Status and data at analgesia initiation			
Cervical dilation, cm	2.1 (0.9); *n* = 2,745	2.2 (0.9); *n* = 1,342	2.1 (0.9); *n* = 1,403
Recorded membrane rupture	1,088/3,391 (32.1%)	529/1,653 (32.0%)	559/1,738 (32.2%)
Pre-analgesia WBC, ×10^9^/L	9.4 (2.5); *n* = 3,096	9.4 (2.5); *n* = 1,517	9.3 (2.5); *n* = 1,579
Pre-analgesia hemoglobin, g/L	122.9 (11.8); *n* = 3,097	122.8 (11.6); *n* = 1,518	123.0 (12.0); *n* = 1,579
Pre-analgesia platelets, ×10^9^/L	205.7 (50.9); *n* = 3,096	205.6 (49.2); *n* = 1,517	205.9 (52.4); *n* = 1,579
Dural puncture epidural	2,091/3,225 (64.8%)	1,035/1,565 (66.1%)	1,056/1,660 (63.6%)
Subsequent course (not baseline)			
Total analgesia duration, hours	10.5 (6.4); *n* = 3,393	9.9 (5.8); *n* = 1,651	11.0 (6.9); *n* = 1,742
Cesarean conversion	211/3,409 (6.2%)	10/1,658 (0.6%)	201/1,751 (11.5%)

SD, standard deviation; WBC, white blood cell.

Continuous data are presented as mean (SD), with available *n*; binary data are *n*/*N* (%), where *N* is the number with an interpretable value. Missing or unknown values equal the column total minus the available denominator. Nulliparity excludes 10 records with missing source parity, and membrane rupture excludes 18 records classified as unknown or other. Recorded complications are linked to diagnosis-list flags; not being listed does not independently verify the absence of disease. Pre-analgesia laboratory values are the nearest available measurements before analgesia, not protocol-timed sampling. Subsequent-course variables are not baseline predictors.

The locked registry-questionnaire export contained no infant identifier or validated mother–infant linkage variable and no validated neonatal outcome fields, including Apgar scores, birth weight, cord blood measurements, resuscitation, neonatal intensive care admission, neonatal infection evaluation, hypoxic–ischemic encephalopathy, or death. Neonatal outcomes were neither inferred nor reconstructed, and no conclusions regarding neonatal safety were drawn.

### 2.4 Primary association analyses

Categorical variables are reported as counts and percentages, and continuous variables as means with standard deviations and medians with interquartile ranges. Observed outcome proportions used Wilson’s 95% confidence intervals (CIs). The study size was determined by the available registry export and questionnaire linkage, and no prospective sample-size calculations were performed.

The strict-fever complete-case model contained 61 events for 11 covariates after calendar-time adjustment was added during revision. To reduce small sample size and separation bias, the primary adjusted analysis used Firth penalized logistic regression with profile penalized-likelihood CIs (15). Firth estimation does not eliminate overfitting or model instability from low event-to-parameter ratios. The any-elevation endpoint used multivariable maximum-likelihood logistic regression. Covariates were entered simultaneously without a stepwise selection. Age was scaled per year, BMI per kg/m^2^, cervical dilation per centimeter, duration per 2 h, pre-analgesia WBC per 1 × 10^9^/L, and calendar time per month. The estimates are associations, not causal effects or validated predictors.

### 2.5 Missing covariates and outcome-linkage sensitivity

The temperature outcomes were not imputed. Complete-case estimates were retained for comparability with the submitted association model and to permit Firth profile penalized-likelihood inference; this does not make the complete-case analysis immune to selection bias. For missing covariates among the 1,658 outcome-linked records, chained-equation multiple imputation generated 50 imputed datasets; predictive mean matching was used for continuous variables and logistic imputation for DPE. The imputation model included both observed temperature outcomes, calendar time, and all the analysis covariates. The estimates were combined using Rubin's rules (16). Complete-case Firth CIs are profile penalized-likelihood intervals, whereas pooled multiple-imputation CIs are Wald intervals combined with Rubin's rules.

Selection related to questionnaire linkage was assessed in all 3,409 records by a direct comparison of linked and unlinked records. A logistic observation model included calendar month, age, BMI, nulliparity, cervical dilation, membrane status, gestational diabetes, hypertensive disorders, DPE or unknown technique, non-linear analgesia duration, pre-analgesia WBC, and cesarean conversion. Median values plus missingness indicators were used only within this observation model. The individual observation-model coefficients were not interpreted. Stabilized inverse probability of outcome observation weighting (IPOW) values were truncated at the 1st and 99th percentiles without clipping the estimated observation probabilities. We report the raw and truncated weight distributions, effective sample size, direct linked-vs.-unlinked differences, weighted balance against the source target, and IPOW-standardized outcome proportions; alternative truncation thresholds were examined (17). The survey intervals were conditional on the estimated observation model and weights. Because only 10 cesarean conversion records had linked outcomes, a restricted analysis targeting records without conversion assessed better overlap conditions. These analyses address selection on measured variables only and cannot remove bias from unmeasured or non-ignorable non-linkage.

### 2.6 Additional sensitivity and descriptive analyses

Supporting analyses included conventional logistic regression for strict fever, models based on 50 imputed datasets, combined multiple-imputation plus IPOW models, repeated-identifier exclusion, and restriction of BMI to 15–50 kg/m^2^. Multiple-imputation-only models used Firth regression for strict fever and conventional logistic regression for any elevation, and combined multiple-imputation plus IPOW models used unpenalized survey-weighted logistic regression for both outcomes. Three repeated hospital identifiers occurred across six source rows, of which one record contributed to the linked complete-case analysis; that record was excluded from the repeated-identifier sensitivity analysis. For any elevation, a *post hoc* three-degree-of-freedom natural-spline duration model was compared with the linear model using a likelihood-ratio test; the *p*-value for non-linearity was nominal and exploratory. A marginal standardized curve used 1,000 record-level bootstrap samples for pointwise 95% CIs.

A separate linkage sensitivity analysis excluded the union of records with multiple matched questionnaires and records whose selected questionnaires had multiple candidate registry matches. Both primary complete-case models were then refitted using the same covariates and estimators. This checks the sensitivity to flagged ambiguities but cannot validate every remaining link.

Post-analgesia WBC count, neutrophil percentage, C-reactive protein (CRP) level, and their changes are summarized only when available. Availability was reported for each temperature group. Spearman correlations with the ordered questionnaire categories were exploratory and were adjusted across eight comparisons using the Benjamini-Hochberg procedure. No laboratory pattern was interpreted as an unbiased, biological dose–response relationship.

### 2.7 Ethics

This retrospective study was approved by the Ethics Committee of Fujian Maternity and Child Health Hospital (approval No. 2026KY023; 27 January 2026) and was conducted in accordance with the Declaration of Helsinki and the applicable institutional requirements. The requirement for written informed consent was waived. Data were handled under institutional confidentiality safeguards, and only aggregated results are reported.

### 2.8 Reproducibility and software

All reported statistical analyses, aggregate tables, and analytic figures in this revision were generated in R version 4.5.2 from the locked analysis export, with upstream questionnaire files used only for linkage provenance and ambiguity sensitivity. The supplied R scripts specify model formulas, 50 imputed datasets, fixed random seeds, figure-generation steps, and focused linkage and missingness audits; package versions, session information, and input/output checksums are provided. The source dataset was not altered. Source denominators and event counts, fitted model estimates, and figure data were cross-checked against the locked-file hash and independently generated aggregate outputs before the manuscript assembly. The revised statistical analysis and reporting were independently reviewed by a statistician.

## 3 Results

### 3.1 Cohort, linkage, and observed temperature outcomes

Of the 3,409 source-registry records, 1,658 (48.6%) had a linked questionnaire temperature outcome, and 1,751 (51.4%) did not (Figure 1).

**Figure 1:**
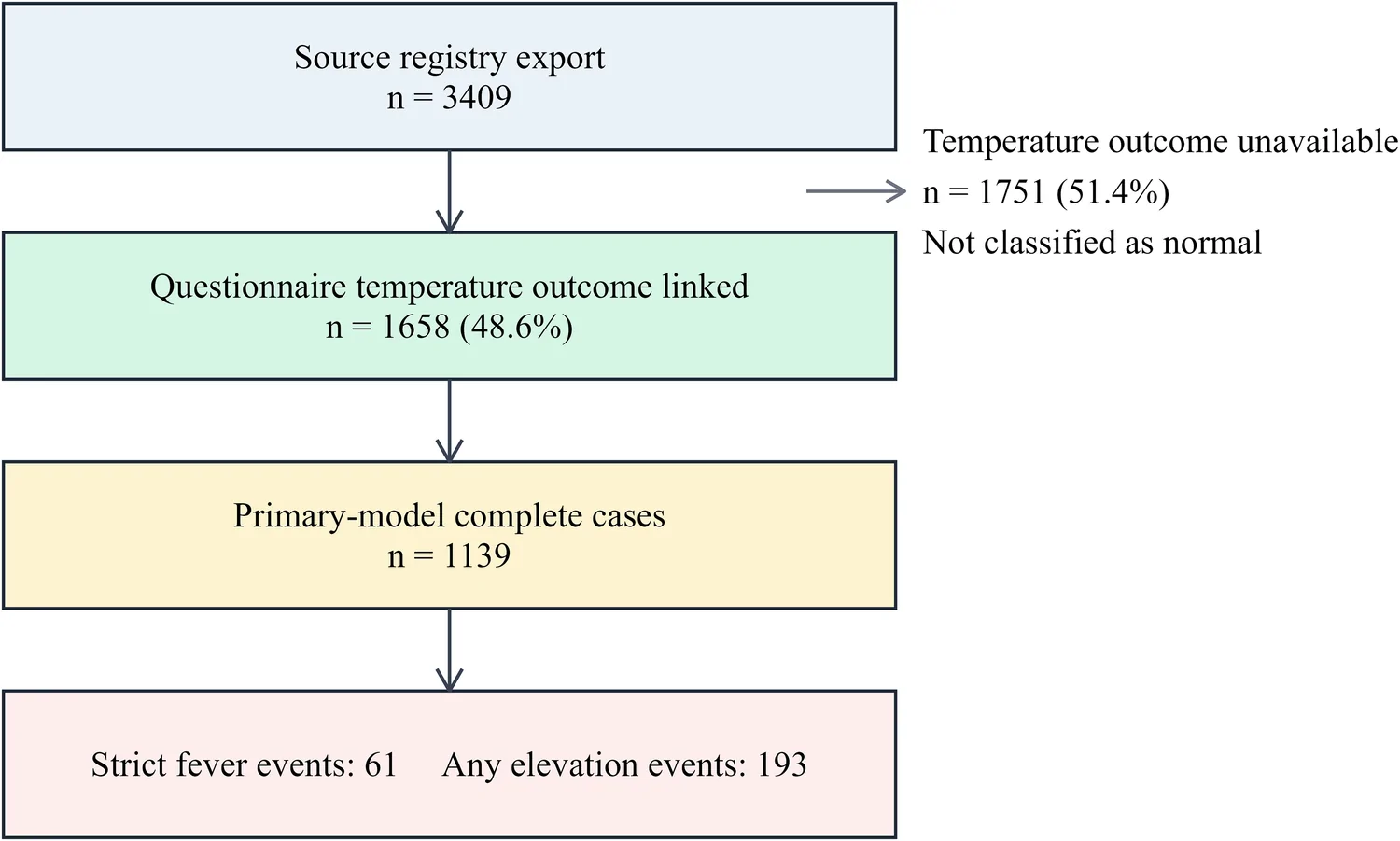
Flow of source-registry records through questionnaire-outcome linkage and complete-case analysis. Records without linked temperature outcomes were not classified as normal. The complete-case counts were applied to both association models.

The grouped maternal and clinical characteristics are presented in Table 1. In the full registry, the mean maternal age was 29.8 years (SD, 4.0) and the mean BMI was 26.0 kg/m^2^ (SD, 3.1). Gestational diabetes and hypertensive disorder were recorded in 626/3,409 (18.4%) and 143/3,409 (4.2%) records, respectively, and the corresponding counts among outcome-linked records were 307/1,658 (18.5%) and 66/1,658 (4.0%).

Among the linked records, 1,371 (82.7%) were classified as normal, 199 (12.0%) as elevated below 38.0 °C, 71 (4.3%) as 38.0–38.5 °C, 15 (0.9%) as 38.6–39.0 °C, and 2 (0.1%) as above 39.0 °C (Table 2). No validated neonatal outcome fields were present in the locked analysis export.

**Table 2:** Distribution of questionnaire-reported maternal temperature categories.

Questionnaire category	*n*	Percent of linked records
Normal temperature	1,371	82.7
Temperature elevation below 38.0 °C	199	12.0
38.0–38.5 °C	71	4.3
38.6–39.0 °C	15	0.9
Above 39.0 °C	2	0.1

Percentages use 1,658 records with linked questionnaire temperature outcomes.

Among the linked records, 6 had more than one matched questionnaire, and 6 had a selected questionnaire with multiple candidate registry matches; 1 record met both criteria, giving 11 flagged records. All linked records had an upstream platform update date, occurring 0–10 calendar days after the registry date; 1,572 (94.8%) were updated after one or two days. These intervals describe the linkage metadata, not a validated recall interval or the time to fever onset.

Strict fever was reported in 88/1,658 linked records (5.3%; 95% CI, 4.3%–6.5%), whereas any elevation was reported in 287/1,658 (17.3%; 95% CI, 15.6%–19.2%). The corresponding IPOW-standardized proportions were 5.4% (95% CI, 4.2%–6.5%) and 17.5% (95% CI, 15.6%–19.4%) (Table 3; Figure 2).

**Table 3:** Observed and IPOW-standardized frequencies of the two nested outcomes.

Outcome	Events	Outcome-linked records	Observed proportion, % (95% CI)	IPOW-standardized proportion, % (95% CI)
Questionnaire-reported fever ≥ 38.0 °C	88	1,658	5.3 (4.3–6.5)	5.4 (4.2–6.5)
Any questionnaire-reported temperature elevation	287	1,658	17.3 (15.6–19.2)	17.5 (15.6–19.4)

CI, confidence interval; IPOW, inverse probability of outcome observation weighting.

The two outcomes are nested and should not be summed.

**Figure 2:**
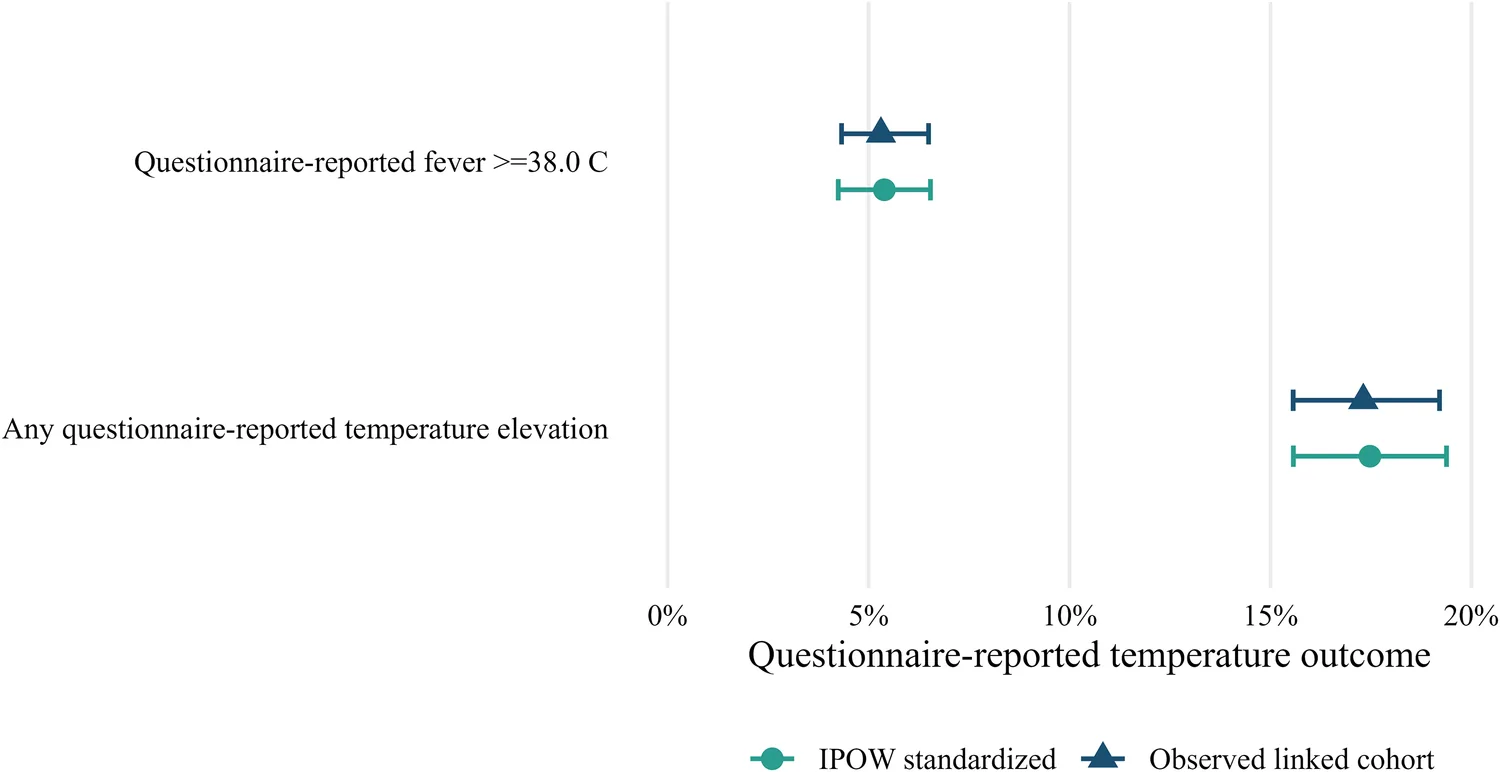
Observed and inverse probability of outcome observation weighted proportions of the two nested questionnaire-derived temperature outcomes. The points represent estimates, and the horizontal lines represent the 95% confidence intervals. Weighting targets the measured covariate distribution of the source-registry but does not address unmeasured or non-ignorable non-linkages.

Largest direct linked-vs.-unlinked differences were observed for cesarean conversion [absolute standardized mean difference (SMD), 0.469], duration (0.178), and calendar time (0.148). Against the source-registry target, the corresponding pre-weighting SMDs were 0.312, 0.096, and 0.075. Raw stabilized weights ranged from 0.642 to 16.258. The 1st/99th-percentile truncation produced weights from 0.686 to 2.170 and an effective sample size of 1,549.4. Most measured characteristics were balanced to an absolute SMD below 0.02, but cesarean conversion remained imbalanced (SMD, 0.254), indicating incomplete positivity and residual selection concern (Supplementary Tables S1,S3; Supplementary Figures S1,S2).

Weight-truncation sensitivity was greater for strict fever: the untruncated standardized estimate was 6.3% (95% CI, 4.1%–8.5%), compared with 5.4% in the primary truncated analysis. The corresponding estimates for any elevation were 18.3% and 17.5%. In the restricted target excluding cesarean conversion, the standardized proportions were 5.2% and 17.2%, and the maximum post-weighting SMD among the 11 reported non-conversion characteristics, evaluated using available values, was 0.012 (Supplementary Tables S3B,S3C). This balance summary excludes missingness indicators: all 16 source records with missing age lacked a linked outcome, including in the restricted target, so weighting could not represent this stratum (Supplementary Table S2).

### 3.2 Complete-case and imputed association analyses

The characteristics by strict-fever status are shown in Table 4. The complete-case models included 1,139 records, 61 strict-fever events, and 193 any-elevation events. Cervical dilation accounted for most of the missing covariates (316/1,658; 19.1%), followed by pre-analgesia WBC (141/1,658; 8.5%) and DPE (93/1,658; 5.6%) (Supplementary Table S2).

**Table 4:** Characteristics of records with and without strict questionnaire-reported fever.

Characteristic	No strict fever (*n* = 1,570)	Strict fever (*n* = 88)
Maternal age, years	29.6 (4.1); 29.0 [27.0–32.0]; *n* = 1,570	29.6 (4.0); 30.0 [27.0–32.0]; *n* = 88
Body mass index, kg/m^2^	25.9 (3.2); 25.7 [23.8–27.7]; *n* = 1,565	26.4 (3.0); 26.1 [24.4–28.2]; *n* = 88
Nulliparity	1,134/1,570 (72.2%)	83/88 (94.3%)
Cervical dilation, cm	2.2 (0.9); 2.0 [2.0–2.0]; *n* = 1,271	2.0 (0.7); 2.0 [2.0–2.0]; *n* = 71
Ruptured membranes	502/1,570 (32.0%)	27/88 (30.7%)
Gestational diabetes	287/1,570 (18.3%)	20/88 (22.7%)
Hypertensive disorder	59/1,570 (3.8%)	7/88 (8.0%)
Dural puncture epidural	989/1,482 (66.7%)	46/83 (55.4%)
Analgesia duration, h	9.8 (5.7); 8.9 [5.1–13.4]; *n* = 1,564	12.4 (5.4); 11.9 [8.8–14.9]; *n* = 87
Pre-analgesia WBC, ×10^9^/L	9.5 (2.5); 9.2 [7.8–10.7]; *n* = 1,435	9.3 (2.2); 9.0 [7.6–10.8]; *n* = 82

BMI, body mass index; IQR, interquartile range; SD, standard deviation; WBC, white blood cell.

Continuous values are presented as mean (SD); median [IQR]; available n. Binary values are expressed as n/N (%). This table retains the locked binary-indicator coding used in the models; unlike Table 1, source parity or membrane entries not recorded as positive remain in the denominators (Section 2.3).

In the Firth model, nulliparity had a large but imprecise association with strict fever [adjusted odds ratio (OR), 8.05; 95% CI, 2.51–40.76]. The DPE estimate (OR, 0.60; 95% CI, 0.35–1.02) and the duration estimate (OR per 2 h, 1.10; 95% CI, 1.00–1.20) both included or approached the null boundary (Table 5). In the Firth analysis based on 50 imputed datasets, the nulliparity estimate attenuated to 5.84 (95% CI, 2.34–14.57), illustrating sensitivity to sparse cells and missing covariates (Supplementary Table S4). Firth estimation reduced the separation bias but did not remove the uncertainty from fitting 11 covariates to 61 events.

**Table 5:** Crude and adjusted associations with strict fever and any temperature elevation.

Outcome	Covariate	Crude OR (95% CI)	Crude *p-*value	Adjusted OR (95% CI)	Adjusted *p-*value
Fever ≥38.0 °C	Maternal age, per year	0.99 (0.93–1.06)	0.837	1.05 (0.98–1.13)	0.190
Fever ≥38.0 °C	Body mass index, per kg/m^2^	1.03 (0.95–1.12)	0.469	1.04 (0.95–1.14)	0.365
Fever ≥38.0 °C	Nulliparity	8.60 (2.93–41.72)	<0.001	8.05 (2.51–40.76)	<0.001
Fever ≥38.0 °C	Cervical dilation, per cm	0.82 (0.56–1.13)	0.248	0.90 (0.60–1.26)	0.580
Fever ≥38.0 °C	Ruptured membranes	0.87 (0.49–1.50)	0.620	0.89 (0.49–1.56)	0.694
Fever ≥38.0 °C	Gestational diabetes	1.49 (0.81–2.61)	0.195	1.41 (0.76–2.50)	0.268
Fever ≥38.0 °C	Hypertensive disorder	1.30 (0.35–3.49)	0.657	0.81 (0.21–2.26)	0.717
Fever ≥38.0 °C	Dural puncture epidural	0.57 (0.34–0.96)	0.034	0.60 (0.35–1.02)	0.060
Fever ≥38.0 °C	Analgesia duration, per 2 h	1.17 (1.08–1.27)	<0.001	1.10 (1.00–1.20)	0.061
Fever ≥38.0 °C	Pre-analgesia WBC, per 1 × 10^9^/L	1.00 (0.89–1.10)	0.977	1.00 (0.89–1.11)	0.980
Fever ≥38.0 °C	Calendar time, per month	0.92 (0.82–1.02)	0.106	0.93 (0.83–1.03)	0.176
Any temperature elevation	Maternal age, per year	1.02 (0.98–1.06)	0.278	1.08 (1.03–1.13)	<0.001
Any temperature elevation	Body mass index, per kg/m^2^	1.02 (0.97–1.07)	0.388	1.02 (0.97–1.08)	0.415
Any temperature elevation	Nulliparity	3.42 (2.11–5.55)	<0.001	3.23 (1.86–5.63)	<0.001
Any temperature elevation	Cervical dilation, per cm	0.79 (0.64–0.97)	0.028	0.86 (0.69–1.07)	0.166
Any temperature elevation	Ruptured membranes	0.89 (0.64–1.24)	0.497	0.91 (0.64–1.29)	0.583
Any temperature elevation	Gestational diabetes	1.02 (0.69–1.50)	0.916	0.96 (0.65–1.44)	0.861
Any temperature elevation	Hypertensive disorder	1.24 (0.61–2.52)	0.557	0.88 (0.42–1.86)	0.739
Any temperature elevation	Dural puncture epidural	0.83 (0.61–1.15)	0.265	0.88 (0.62–1.23)	0.445
Any temperature elevation	Analgesia duration, per 2 h	1.18 (1.12–1.24)	<0.001	1.13 (1.06–1.20)	<0.001
Any temperature elevation	Pre-analgesia WBC, per 1 × 10^9^/L	1.03 (0.97–1.09)	0.316	1.04 (0.97–1.11)	0.266
Any temperature elevation	Calendar time, per month	0.89 (0.83–0.95)	<0.001	0.88 (0.82–0.95)	<0.001

CI, confidence interval; OR, odds ratio; WBC, white blood cell.

The strict-fever model uses Firth penalized logistic regression with profile penalized-likelihood confidence intervals, and the any-elevation model uses maximum-likelihood logistic regression. Both models use 1,139 complete records (61 and 193 events, respectively) and include all listed covariates simultaneously.

For any elevation, age (adjusted OR per year, 1.08; 95% CI, 1.03–1.13), nulliparity (OR, 3.23; 95% CI, 1.86–5.63), and duration (OR per 2 h, 1.13; 95% CI, 1.06–1.20) were associated with higher odds, whereas calendar time was inversely associated (OR per month, 0.88; 95% CI, 0.82–0.95). The duration OR summarizes the working linear model, and does not imply a constant effect across the observed duration range. Sensitivity estimates based on 50 imputed datasets and combined imputation plus IPOW were directionally similar for these four covariates; strict-fever estimates for age, BMI, duration, and calendar time were less stable across methods (Supplementary Table S4).

### 3.3 Duration pattern

Analgesia duration distributions overlapped substantially but shifted toward longer values among records with reported elevations (Figure 3). The *post hoc* natural-spline model for any elevation fits better than the linear-duration model (likelihood-ratio chi-square = 12.33; 2 df; nominal exploratory *p* for non-linearity = 0.002), with the marginal standardized curve rising through approximately 12 h and then flattening (Figure 4; Supplementary Table S5). Because the total duration was not anchored to temperature onset or labor duration, this pattern cannot establish whether longer analgesia preceded or caused the reported elevation. Duration may instead indicate longer or more complicated labor, and residual confounding by labor duration and reverse temporal relationships cannot be resolved.

**Figure 3:**
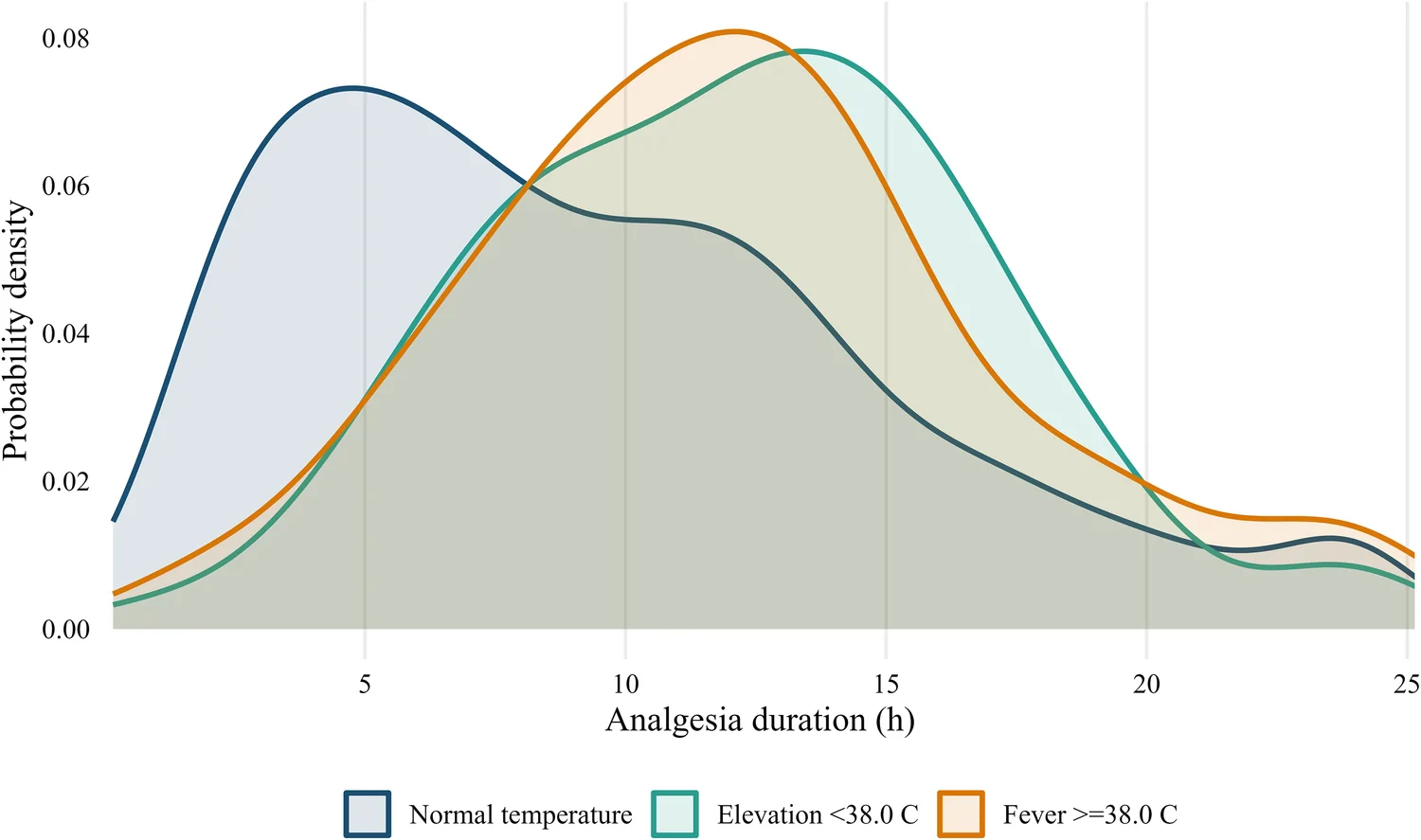
Probability-density distributions of total analgesia duration across the three questionnaire-derived temperature groups. The plot shows overlapping distributions and is descriptive because the temperature-onset timing was unavailable.

**Figure 4:**
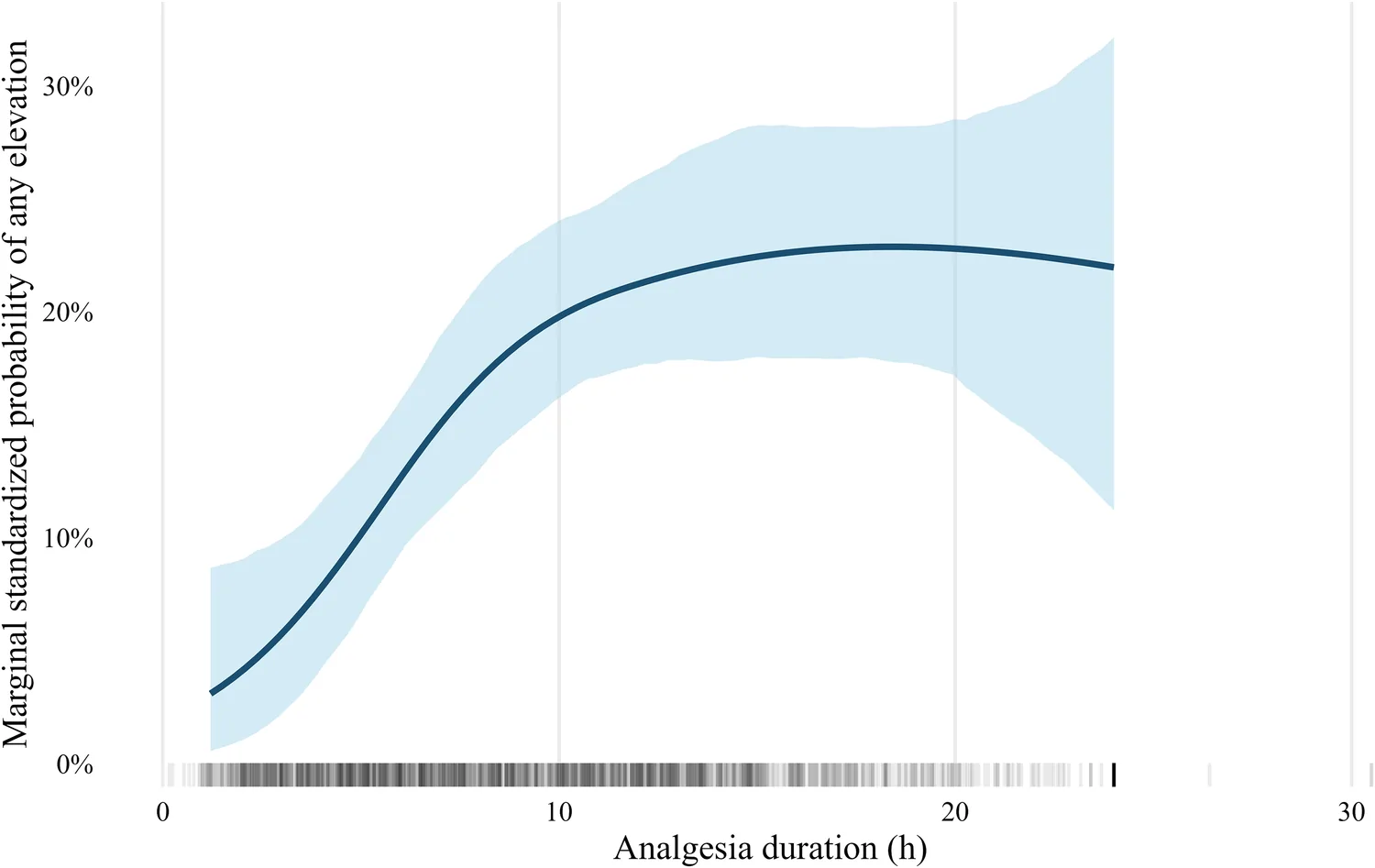
Marginal standardized probability of any questionnaire-reported temperature elevation across total analgesia duration from the *post hoc* three-degree-of-freedom natural-spline model. The band is a pointwise 95% record-level bootstrap interval based on 1,000 resamples. The duration was not anchored to the temperature onset; therefore, the curve does not establish temporal order or causality.

### 3.4 Laboratory ascertainment and other sensitivity analyses

Post-analgesia testing was differential. WBC was available in 153/1,371 normal-temperature records (11.2%), 142/199 records with elevations below 38.0 °C (71.4%), and 80/88 strict-fever records (90.9%). CRP availability was 5.9%, 36.2%, and 50.0%, respectively (Supplementary Table S6; Supplementary Figure S3).

Several ordinal correlations remained below the Benjamini–Hochberg false-discovery threshold, but they used clinically selected, variable-specific subsets. Changes in CRP included only 46 records and did not remain below *q* = 0.05. These correlations were reported as descriptive signals rather than biological gradients (Supplementary Table S7). Excluding the one linked complete-case record with a repeated source hospital identifier or restricting BMI to 15–50 kg/m^2^ did not materially change the selected association estimates (Supplementary Table S8).

Excluding all 11 flagged questionnaire links retained 1,647 linked records, 88 strict-fever events, and 285 any-elevation events. The corresponding complete-case models retained 1,131 records, 61 strict-fever events, and 193 any-elevation events. The estimates were numerically similar to those of the primary models: the strict-fever nulliparity OR was 8.02 (95% CI, 2.51–40.62), and the any-elevation duration OR per 2 h was 1.13 (95% CI, 1.07–1.20) (Supplementary Tables S9A,S9B). This sensitivity result does not exclude unrecognized linkage errors.

## 4 Discussion

The main finding was measurement-related: the observed proportion was 5.3% under the strict threshold and 17.3% under the broader questionnaire category. Primary truncated selection weighting changed these values only modestly, but the untruncated strict-fever estimate was higher, and weighting could not balance cesarean conversion because questionnaire-outcome overlap was poor. Association estimates were also definition-sensitive, and the strict-fever results remained imprecise despite bias-reduced regression. In particular, the large nulliparity OR and its wide interval may reflect sparse cells and model instability, and the Firth estimation does not establish the magnitude of this association with precision. These features support an exploratory hypothesis-generating interpretation rather than causal or predictive claims.

An early prospective study documented increasing maternal temperature during epidural labor analgesia in otherwise afebrile women without clinical evidence of infection (18). Retrospective cohort evidence supports an association between epidural exposure and maternal hyperthermia but does not establish neuraxial analgesia as the cause of every febrile episode (19). The failure of prophylactic antibiotics to prevent epidural-related fever in a randomized trial supports a non-infectious pathway in at least some patients (20). Inflammatory-marker observations in prior work remain biologically plausible, but the selected testing in our dataset precludes comparable mechanistic inferences (21).

Mechanistic evidence remains unclear. A 2023 letter proposed examining fetal immune function and *in utero* temperature to improve the understanding of epidural-related maternal fever (22). A recent multicenter Mendelian randomization study found no evidence that genetically predicted interleukin-1 receptor antagonist levels altered the likelihood of maternal fever or intrapartum antibiotic administration in women receiving epidural analgesia (23). Experimental ropivacaine data identified possible inflammatory signaling mechanisms but could not be mapped directly onto the associations observed in our registry (24). Thermoregulatory mechanisms also remain plausible, including cutaneous sympathetic blockade during epidural analgesia (25).

Clinical interpretation must distinguish between temperature observations and etiology because infectious and non-infectious causes overlap (26). Contemporary descriptions of clinical chorioamnionitis emphasize that fever alone is not an etiological diagnosis (27). The terminology of intrauterine inflammation or infection, or both, was developed partly to reduce the automatic equivalence between intrapartum fever and chorioamnionitis (28).

Antibiotic administration for isolated maternal fever is associated with differences in maternal and neonatal management and outcomes (29). A retrospective cohort study found different maternal and neonatal infectious outcomes between isolated intrapartum fever and clinical chorioamnionitis (30). The relationship between the duration and magnitude of hyperthermia and neonatal encephalopathy underscores the need for continuous, timestamped measurements in future cohorts (31).

Our calendar-adjusted DPE estimate should not be interpreted as a protective factor. The randomized evidence for preventive and therapeutic strategies for epidural-related maternal fever remains heterogeneous (32). A randomized DPE comparison primarily evaluated analgesic quality rather than fever (33). In parturients with obesity receiving programmed intermittent boluses, a randomized trial found no significant improvement in the prespecified composite analgesia-quality outcome with DPE over standard epidural analgesia (34). Recent dose-finding comparisons have also focused on analgesia initiation rather than maternal temperature (35). A 2025 meta-analysis focused on analgesic and block-quality outcomes but did not evaluate maternal fever and therefore could not resolve fever risk (36).

Indirect evidence from a matched retrospective study showed no significant difference in intrapartum fever between continuous spinal and epidural labor analgesia; however, this was not a DPE comparison (37). A contemporary review evaluated modern labor epidural approaches across analgesic, obstetric, maternal, and fetal outcomes, which is a broader scope than the temperature categories available in our registry (38).

The linkage analysis showed modest differences for most measured variables but a major deficit of questionnaire outcomes after cesarean conversion. Combining multiple imputations with observation weighting can address the measured components of missingness but cannot recover unrecorded outcomes or guarantee validity under non-ignorable selection (39). The close primary-truncated and unweighted proportions apply only to the fitted observation model; the dependence of the strict-fever estimate on weight truncation and the conditional treatment of estimated weights preclude their interpretation as proof that selection bias is absent.

For clinical quality improvement, units should standardize the measurement device and anatomical site, record the actual value and timestamp, repeat abnormal measurements, and document maternal vital signs, fetal status, infection risk factors, and concurrent interventions. A rise in maternal temperature should not be automatically attributed to epidural analgesia. Assessment should follow local multidisciplinary obstetric, anesthetic, infectious disease, and neonatal pathways, and treatment indications and responses should be recorded. Audits should track questionnaire linkage, temperature-record completeness, laboratory-testing indications, escalation decisions, and maternal and neonatal outcomes. These are process recommendations derived from the study's measurement gaps, not interventions tested by this dataset.

Several limitations directly constrain the interpretation. More than half of the source records lacked a linked temperature outcome, complete registry-level reasons for non-linkage were unavailable, IPOW could not overcome poor cesarean-conversion overlap or the absence of outcomes in the age-missing stratum, and the strict-fever estimate varied with weight truncation. The IPOW intervals were conditional on the fitted observation model. Deterministic name–age–date matching may leave unrecognized linkage errors even after excluding flagged ambiguities. Although the original question and platform update timestamps were recovered, completion procedures, measurement sources, continuous values, and clinical timestamps remained unverified, and recall or reporting variability cannot be excluded. The total duration and post-analgesia laboratory measurements had uncertain ordering relative to the temperature onset. Laboratory testing was strongly selected based on clinical concerns, treatment assignment was non-random, and strict-fever events were sparse. Pre-export eligibility could not be reconstructed, and single-center practice limits the generalizability.

Neonatal outcomes are an additional important limitation. The locked export did not contain infant identifiers or validated neonatal fields; therefore, the study could not evaluate neonatal status at birth or the safety consequences of maternal temperature elevation. A valid extension would require a prespecified mother–infant linkage, explicit handling of multiple births, neonatal outcome definitions, and outcome adjudication. The present analysis is restricted to observed maternal outcome frequency, selection diagnostics, and exploratory associations, and it does not develop or validate a clinical prediction model.

## 5 Conclusion

Among the records with a linked questionnaire outcome, the observed frequency of questionnaire-reported maternal temperature elevation varied substantially between the strict and broader definitions IPOW only changed the point estimates modestly; however, its diagnostics revealed limited outcome overlap after cesarean conversion. Sparse strict-fever events, missing covariates, uncertain timing, and differential laboratory testing preclude causal, predictive, and neonatal-safety conclusions. Prospective studies should use continuous timestamped temperature measurements, standardized clinical adjudication, complete mother–infant linkage, and prespecified maternal and neonatal outcomes.

## Data Availability

The datasets presented in this article are not readily available because they contain potentially identifiable maternal health information and are subject to institutional privacy and ethics restrictions. Requests will be considered subject to approval by the Fujian Maternity and Child Health Hospital and its ethics committee. The R code and aggregate reproducibility outputs for this revision are provided in the Supplementary Material. Requests for access to the datasets should be directed to Shihong Huang (huangshihong@fjsfy.com).

## References

[1] Lieberman E Lang JM Frigoletto F Richardson DK Ringer SA Cohen A. Epidural analgesia, intrapartum fever, and neonatal sepsis evaluation. Pediatrics. (1997) 99(3):415–9. 10.1542/peds.99.3.415 9041298

[2] Greenwell EA Wyshak G Ringer SA Johnson LC Rivkin MJ Lieberman E. Intrapartum temperature elevation, epidural use, and adverse outcome in term infants. Pediatrics. (2012) 129(2):e447–54. 10.1542/peds.2010-2301 22291120 PMC3357045

[3] Lieberman E Lang J Richardson DK Frigoletto FD Heffner LJ Cohen A. Intrapartum maternal fever and neonatal outcome. Pediatrics. (2000) 105(1 Pt 1):8–13. 10.1542/peds.105.1.8 10617697

[4] Philip J Alexander JM Sharma SK Leveno KJ McIntire DD Wiley J. Epidural analgesia during labor and maternal fever. Anesthesiology. (1999) 90(5):1271–5. 10.1097/00000542-199905000-00008 10319772

[5] Segal S. Labor epidural analgesia and maternal fever. Anesth Analg. (2010) 111(6):1467–75. 10.1213/ANE.0b013e3181f713d4 20861420

[6] Sultan P David AL Fernando R Ackland GL. Inflammation and epidural-related maternal fever: proposed mechanisms. Anesth Analg. (2016) 122(5):1546–53. 10.1213/ANE.0000000000001195 27101499

[7] del Arroyo AG Sanchez J Patel S Phillips S Reyes A Cubillos C. Role of leucocyte caspase-1 activity in epidural-related maternal fever: a single-centre, observational, mechanistic cohort study. Br J Anaesth. (2019) 122(1):92–102. 10.1016/j.bja.2018.09.024 30579413

[8] Riley LE Celi AC Onderdonk AB Roberts DJ Johnson LC Tsen LC. Association of epidural-related fever and noninfectious inflammation in term labor. Obstet Gynecol. (2011) 117(3):588–95. 10.1097/AOG.0b013e31820b0503 21343762

[9] Sultan P Segal S. Epidural-related maternal fever: still a hot topic, but what are the burning issues? Anesth Analg. (2020) 130(2):318–20. 10.1213/ANE.0000000000004576 31934906

[10] Patel S Ciechanowicz S Blumenfeld YJ Sultan P. Epidural-related maternal fever: incidence, pathophysiology, outcomes, and management. Am J Obstet Gynecol. (2023) 228(5S):S1283–S1304.e1. 10.1016/j.ajog.2022.06.026 36925412

[11] Attard Cortis P Bleeser T Devroe S Lucas N. Neuraxial labour analgesia and maternal fever: a narrative review. Eur J Anaesthesiol. (2026) 43(1):45–52. 10.1097/EJA.0000000000002280 41017360

[12] Hensel D Zhang F Carter EB Frolova AI Odibo AO Kelly JC. Severity of intrapartum fever and neonatal outcomes. Am J Obstet Gynecol. (2022) 227(3):513.e1–e8. 10.1016/j.ajog.2022.05.031 35598690

[13] Cartledge A Hind D Bradburn M Martyn-St James M Davenport S Tung WS. Interventions for the prevention or treatment of epidural-related maternal fever: a systematic review and meta-analysis. Br J Anaesth. (2022) 129(4):567–80. 10.1016/j.bja.2022.06.022 35934529 PMC9575042

[14] Von Elm E Altman DG Egger M Pocock SJ Gøtzsche PC Vandenbroucke JP. The Strengthening the Reporting of Observational Studies in Epidemiology (STROBE) statement: guidelines for reporting observational studies. J Clin Epidemiol. (2008) 61(4):344–9. 10.1016/j.jclinepi.2007.11.008 18313558

[15] Heinze G Schemper M. A solution to the problem of separation in logistic regression. Stat Med. (2002) 21(16):2409–19. 10.1002/sim.1047 12210625

[16] White IR Royston P Wood AM. Multiple imputation using chained equations: issues and guidance for practice. Stat Med. (2011) 30(4):377–99. 10.1002/sim.4067 21225900

[17] Seaman SR White IR. Review of inverse probability weighting for dealing with missing data. Stat Methods Med Res. (2013) 22(3):278–95. 10.1177/0962280210395740 21220355

[18] Fusi L Steer PJ Maresh MJ Beard RW. Maternal pyrexia associated with the use of epidural analgesia in labour. Lancet. (1989) 333(8649):1250–2. 10.1016/s0140-6736(89)92341-6. PMID 2566791. 2566791

[19] Yancey MK Zhang J Schwarz J Dietrich CS Klebanoff M. Labor epidural analgesia and intrapartum maternal hyperthermia. Obstet Gynecol. (2001) 98(5 Pt 1):763–70. 10.1016/s0029-7844(01)01537-x 11704166

[20] Sharma SK Rogers BB Alexander JM McIntire DD Leveno KJ. A randomized trial of the effects of antibiotic prophylaxis on epidural-related fever in labor. Anesth Analg. (2014) 118(3):604–10. 10.1213/ANE.0b013e3182a5d539 24299929

[21] Goetzl L Evans T Rivers J Suresh MS Lieberman E. Elevated maternal and fetal serum interleukin-6 levels are associated with epidural fever. Am J Obstet Gynecol. (2002) 187(4):834–8. 10.1067/mob.2002.127135 12388959

[22] Sultan P Blumenfeld Y. Elucidating the effects of labor analgesia on fetal immune function and in utero temperature: a novel approach to understanding epidural-related maternal fever. Am J Obstet Gynecol. (2023) 229(4):476–7. 10.1016/j.ajog.2023.04.035 37120048

[23] Ali A Noblett J Usman N Wray S Blake H Gutierrez del Arroyo A. Interleukin-1 receptor antagonist polymorphisms in women receiving epidural analgesia who develop maternal intrapartum fever: a prospective, multicentre Mendelian randomised study. Br J Anaesth. (2025) 135(1):134–40. 10.1016/j.bja.2025.03.037 40368685 PMC12226748

[24] Wohlrab P Boehme S Kaun C Wojta J Spittler A Saleh L. Ropivacaine activates multiple proapoptotic and inflammatory signaling pathways that might subsume to trigger epidural-related maternal fever. Anesth Analg. (2020) 130(2):321–31. 10.1213/ANE.0000000000004402 31498191

[25] Iacona GMV Rolph AR Manteigas HFM Strutton PH Low DA Mullington CJ. The cutaneous sympathetic blockade associated with labour epidural analgesia: a quasi-experimental study conducted during labour and after delivery. Br J Anaesth. (2025) 135(5):1231–9. 10.1016/j.bja.2025.07.077 40930874 PMC12597345

[26] Goetzl L. Maternal fever in labor: etiologies, consequences, and clinical management. Am J Obstet Gynecol. (2023) 228(5S):S1274–82. 10.1016/j.ajog.2022.11.002 36997396

[27] Jung E Romero R Suksai M Gotsch F Chaemsaithong P Erez O. Clinical chorioamnionitis at term: definition, pathogenesis, microbiology, diagnosis, and treatment. Am J Obstet Gynecol. (2024) 230(3S):S807–40. 10.1016/j.ajog.2023.02.002 38233317 PMC11288098

[28] Higgins RD Saade G Polin RA Grobman WA Buhimschi IA Watterberg K. Evaluation and management of women and newborns with a maternal diagnosis of chorioamnionitis: summary of a workshop. Obstet Gynecol. (2016) 127(3):426–36. 10.1097/AOG.0000000000001246 26855098 PMC4764452

[29] Bank TC Nuss E Subedi K Hoffman MK Sciscione A. Outcomes associated with antibiotic administration for isolated maternal fever in labor. Am J Obstet Gynecol. (2022) 226(2):255.e1–e7. 10.1016/j.ajog.2021.07.020 34363779 PMC8810660

[30] Abu Shqara R Glikman D Jad S Rechnitzer H Lowenstein L Frank Wolf M. Antibiotic treatment of women with isolated intrapartum fever vs clinical chorioamnionitis: maternal and neonatal outcomes. Am J Obstet Gynecol. (2023) 229(5):540.e1–e9. 10.1016/j.ajog.2023.05.013 38051599

[31] Cornet MC Kuzniewicz MW Scheffler AW Garabedian C Gaw SL Wu YW. Maternal fever during labor and the risk of neonatal encephalopathy: duration and magnitude of hyperthermia. Am J Obstet Gynecol. (2025) 233(6):651.e1–651.e12. 10.1016/j.ajog.2025.07.046 40754173 PMC12412422

[32] Yu R Deng X Hu N Chen DX. Comparative effectiveness of diverse strategies for the prevention or treatment of epidural-related maternal fever: a systematic review and network meta-analysis of randomized controlled trials. Am J Obstet Gynecol. (2025) 233(6):581–590.e30. 10.1016/j.ajog.2025.07.029 40712861

[33] Chau A Bibbo C Huang C-C Elterman KG Cappiello EC Robinson JN. Dural puncture epidural technique improves labor analgesia quality with fewer side effects compared with epidural and combined spinal epidural techniques: a randomized clinical trial. Anesth Analg. (2017) 124(2):560–9. 10.1213/ANE.0000000000001798 28067707

[34] Tan HS Reed SE Mehdiratta JE Diomede OI Landreth R Gatta LA. Quality of labor analgesia with dural puncture epidural versus standard epidural technique in obese parturients: a double-blind randomized controlled study. Anesthesiology. (2022) 136(5):678–87. 10.1097/ALN.0000000000004137 35157756

[35] Maeda A Villela-Franyutti D Lumbreras-Marquez MI Murthy A Fields KG Justice S. Labor analgesia initiation with dural puncture epidural versus conventional epidural techniques: a randomized biased-coin sequential allocation trial to determine the effective dose for 90% of patients of bupivacaine. Anesth Analg. (2024) 138(6):1205–14. 10.1213/ANE.0000000000006691 37824436

[36] Singh PM Monks DT Bhat AD Borle A Kaur M Yang P. Epidural analgesia versus dural puncture epidural analgesia in labouring parturients: a meta-analysis of randomised controlled trials. Br J Anaesth. (2025) 134(5):1402–14. 10.1016/j.bja.2025.01.033 40121178 PMC12106887

[37] Seiler FA Scavone BM Shahul S Arnolds DE. Maternal fever associated with continuous spinal versus epidural labor analgesia: a single-center retrospective study. Anesth Analg. (2022) 135(6):1153–8. 10.1213/ANE.0000000000005905 35051951

[38] Callahan EC Lee W Aleshi P George RB. Modern labor epidural analgesia: implications for labor outcomes and maternal-fetal health. Am J Obstet Gynecol. (2023) 228(5S):S1260–9. 10.1016/j.ajog.2022.06.017 37164496

[39] Thaweethai T Mukherjee R Arterburn D Fischer H Lee C Shortreed SM. Adjusting for selection bias due to missing data in electronic health records-based research by blending multiple imputation and inverse probability weighting. Stat Med. (2025) 44(15–17):e70151. 10.1002/sim.70151 40662472 PMC13262754

