# Supplementary material for: Definition dependence of questionnaire-reported maternal temperature elevation in a retrospective registry cohort of women receiving neuraxial labor analgesia
Source: Front Med (Lausanne). 2026 Oct 1;13:1960868. doi: 10.3389/fmed.2026.1960868 (PMC13669197; doi:10.3389/fmed.2026.1960868)

Supplementary material

Manuscript ID 1960868 | Definition dependence of questionnaire-reported maternal temperature elevation in a retrospective registry cohort of women receiving neuraxial labor analgesia

Supplementary Table 1. Direct comparison of outcome-linked and outcome-unlinked records and weighted comparison with the source registry target.

Characteristic	Source registry	Outcome-linked	Outcome-unlinked	Absolute SMD linked vs unlinked	Absolute SMD before IPOW	IPOW-standardized linked	Absolute SMD after IPOW
Maternal age, yr	29.8 (4.0); n=3393	29.6 (4.0); n=1658	29.9 (3.9); n=1735	0.075	0.038	29.8 (4.0); n=1658	0.004
Body mass index, kg/m²	26.0 (3.1); n=3388	25.9 (3.1); n=1653	26.2 (3.1); n=1735	0.085	0.044	26.0 (3.1); n=1653	0.016
Nulliparity	2553/3409 (74.9%)	1217/1658 (73.4%)	1336/1751 (76.3%)	0.067	0.034	74.3%; n=1658	0.013
Cervical dilation, cm	2.1 (0.9); n=2745	2.2 (0.9); n=1342	2.1 (0.9); n=1403	0.092	0.047	2.1 (0.9); n=1342	0.016
Ruptured membranes	1088/3409 (31.9%)	529/1658 (31.9%)	559/1751 (31.9%)	0.000	0.000	31.7%; n=1658	0.004
Gestational diabetes	626/3409 (18.4%)	307/1658 (18.5%)	319/1751 (18.2%)	0.008	0.004	18.6%; n=1658	0.007
Hypertensive disorder	143/3409 (4.2%)	66/1658 (4.0%)	77/1751 (4.4%)	0.021	0.011	4.3%; n=1658	0.007
Dural puncture epidural	2091/3225 (64.8%)	1035/1565 (66.1%)	1056/1660 (63.6%)	0.053	0.027	65.2%; n=1565	0.008
Analgesia duration, h	10.5 (6.4); n=3393	9.9 (5.8); n=1651	11.0 (6.9); n=1742	0.178	0.096	10.4 (6.2); n=1651	0.008
Pre-analgesia WBC, x10^9/L	9.4 (2.5); n=3096	9.4 (2.5); n=1517	9.3 (2.5); n=1579	0.052	0.026	9.4 (2.4); n=1517	0.004
Cesarean conversion	211/3409 (6.2%)	10/1658 (0.6%)	201/1751 (11.5%)	0.469	0.312	1.4%; n=1658	0.254

**Characteristic**	**Source registry**	**Outcome-linked**	**Outcome-unlinked**	**Absolute SMD linked vs unlinked**	**Absolute SMD before IPOW**	**IPOW-standardized linked**	**Absolute SMD after IPOW**
Calendar time, months from study start	4.9 (2.4); n=3409	4.7 (2.5); n=1658	5.0 (2.4); n=1751	0.148	0.075	4.9 (2.5); n=1658	0.007

SMD, standardized mean difference; IPOW, inverse probability of outcome observation weighting.

Supplementary Table 2. Variable-level missingness in the source registry and outcome-linked cohort.

Variable	Source registry missing, n/N (%)	Outcome-linked missing, n/N (%)
Questionnaire temperature outcome	1751/3409 (51.4%)	0/1658 (0.0%)
Maternal age	16/3409 (0.5%)	0/1658 (0.0%)
Body mass index	21/3409 (0.6%)	5/1658 (0.3%)
Nulliparity	0/3409 (0.0%)	0/1658 (0.0%)
Cervical dilation	664/3409 (19.5%)	316/1658 (19.1%)
Ruptured membranes	0/3409 (0.0%)	0/1658 (0.0%)
Gestational diabetes	0/3409 (0.0%)	0/1658 (0.0%)
Hypertensive disorder	0/3409 (0.0%)	0/1658 (0.0%)
Dural puncture epidural	184/3409 (5.4%)	93/1658 (5.6%)
Analgesia duration	16/3409 (0.5%)	7/1658 (0.4%)
Pre-analgesia WBC	313/3409 (9.2%)	141/1658 (8.5%)
Calendar time	0/3409 (0.0%)	0/1658 (0.0%)

The questionnaire outcome itself was not imputed. Complete-case association models retained 1,139 records.

Coding clarification for the second revision: missingness of nulliparity and membrane rupture in this table refers to the locked derived model indicators. The source parity field is missing in 10 registry records (1 outcome-linked), and source membrane status is unknown or other in 18 records (5 outcome-linked). Main Table 1 excludes these entries from available-value denominators; the model-based and weighting summaries retain the locked not-positive coding. No model estimates have been changed.

Supplementary Table 3. Observation-weight diagnostics and covariate balance.

Metric	Value
Source registry records	3409
Outcome-linked records	1658
Observed linkage proportion	0.486
Minimum fitted linkage probability	1.90e-07
Maximum fitted linkage probability	0.758
Minimum raw stabilized weight	0.642
Maximum raw stabilized weight	16.258
1st-percentile truncation point	0.686
99th-percentile truncation point	2.170
Minimum truncated weight	0.686
Maximum truncated weight	2.170
Effective sample size after IPOW	1549.4
Maximum absolute SMD before IPOW	0.312
Maximum absolute SMD after IPOW	0.254

Weights were stabilized and truncated at the 1st and 99th percentiles.

Supplementary Table 3B. Restricted-target IPOW sensitivity among records without cesarean conversion.

Target	Outcome	Source records	Linked records	Events	Observed proportion, %	IPOW proportion, % (95% CI)	IPOW effective sample size	Maximum absolute SMD after IPOW
Records without cesarean conversion	Questionnaire-reported fever >=38.0 C	3198	1648	86	5.2	5.2 (4.1-6.3)	1557.8	0.012
Records without cesarean conversion	Any questionnaire-reported temperature elevation	3198	1648	283	17.2	17.2 (15.3-19.0)	1557.8	0.012

The maximum SMD summarizes the 11 reported non-conversion characteristics using available values; it excludes missingness indicators. All 16 source records with missing age also belonged to this restricted target and none had a linked outcome. Restriction does not recover outcomes for converted records or address zero linkage in the age-missing stratum.

Supplementary Table 3C. Sensitivity of IPOW estimates to alternative weight-truncation choices.

Truncation	Outcome	Lower weight bound	Upper weight bound	Effective sample size	IPOW proportion, % (95% CI)
	Questionnaire-reported fever >=38.0 C	0.642	16.258	977.9	6.3 (4.1-8.5)
	Any questionnaire-reported temperature elevation	0.642	16.258	977.9	18.3 (15.6-21.0)
0.5th/99.5th percentiles	Questionnaire-reported fever >=38.0 C	0.679	6.003	1352.4	5.8 (4.3-7.3)
0.5th/99.5th percentiles	Any questionnaire-reported temperature elevation	0.679	6.003	1352.4	18.1 (15.9-20.3)
1st/99th percentiles (primary)	Questionnaire-reported fever >=38.0 C	0.686	2.170	1549.4	5.4 (4.2-6.5)
1st/99th percentiles (primary)	Any questionnaire-reported temperature elevation	0.686	2.170	1549.4	17.5 (15.6-19.4)
2.5th/97.5th percentiles	Questionnaire-reported fever >=38.0 C	0.700	1.544	1578.9	5.3 (4.2-6.4)
2.5th/97.5th percentiles	Any questionnaire-reported temperature elevation	0.700	1.544	1578.9	17.4 (15.5-19.3)

Intervals are conditional on the estimated observation model and weights. The untruncated and three truncated specifications quantify sensitivity to this choice.

Supplementary Table 4. Complete-case, multiple-imputation, and combined multiple-imputation-plus-IPOW association estimates.

Outcome	Method	Covariate	Adjusted OR (95% CI)	p value	N	Events
Fever >=38.0 C	Complete-case Firth (primary)	Maternal age, per yr	1.05 (0.98-1.13)	0.190	1139	61
Fever >=38.0 C	Complete-case Firth (primary)	Body mass index, per kg/m²	1.04 (0.95-1.14)	0.365	1139	61
Fever >=38.0 C	Complete-case Firth (primary)	Nulliparity	8.05 (2.51-40.76)	<0.001	1139	61
Fever >=38.0 C	Complete-case Firth (primary)	Cervical dilation, per cm	0.90 (0.60-1.26)	0.580	1139	61
Fever >=38.0 C	Complete-case Firth (primary)	Ruptured membranes	0.89 (0.49-1.56)	0.694	1139	61
Fever >=38.0 C	Complete-case Firth (primary)	Gestational diabetes	1.41 (0.76-2.50)	0.268	1139	61
Fever >=38.0 C	Complete-case Firth (primary)	Hypertensive disorder	0.81 (0.21-2.26)	0.717	1139	61
Fever >=38.0 C	Complete-case Firth (primary)	Dural puncture epidural	0.60 (0.35-1.02)	0.060	1139	61
Fever >=38.0 C	Complete-case Firth (primary)	Analgesia duration, per 2 h	1.10 (1.00-1.20)	0.061	1139	61
Fever >=38.0 C	Complete-case Firth (primary)	Pre-analgesia WBC, per 1 × 10^9/L	1.00 (0.89-1.11)	0.980	1139	61
Fever >=38.0 C	Complete-case Firth (primary)	Calendar time, per month	0.93 (0.83-1.03)	0.176	1139	61
Fever >=38.0 C	Complete-case maximum likelihood	Maternal age, per yr	1.05 (0.98-1.13)	0.181	1139	61
Fever >=38.0 C	Complete-case maximum likelihood	Body mass index, per kg/m²	1.04 (0.95-1.14)	0.356	1139	61

Outcome	Method	Covariate	Adjusted OR (95% CI)	p value	N	Events
Fever >=38.0 C	Complete-case maximum likelihood	Nulliparity	10.14 (2.32-44.41)	0.002	1139	61
Fever >=38.0 C	Complete-case maximum likelihood	Cervical dilation, per cm	0.87 (0.60-1.27)	0.485	1139	61
Fever >=38.0 C	Complete-case maximum likelihood	Ruptured membranes	0.87 (0.49-1.56)	0.642	1139	61
Fever >=38.0 C	Complete-case maximum likelihood	Gestational diabetes	1.39 (0.76-2.55)	0.281	1139	61
Fever >=38.0 C	Complete-case maximum likelihood	Hypertensive disorder	0.71 (0.21-2.42)	0.581	1139	61
Fever >=38.0 C	Complete-case maximum likelihood	Dural puncture epidural	0.59 (0.35-1.02)	0.057	1139	61
Fever >=38.0 C	Complete-case maximum likelihood	Analgesia duration, per 2 h	1.10 (1.00-1.21)	0.058	1139	61
Fever >=38.0 C	Complete-case maximum likelihood	Pre-analgesia WBC, per 1 × 10^9/L	0.99 (0.89-1.11)	0.905	1139	61
Fever >=38.0 C	Complete-case maximum likelihood	Calendar time, per month	0.92 (0.83-1.03)	0.167	1139	61
Fever >=38.0 C	Multiple imputation (50 datasets), Firth	Maternal age, per yr	1.06 (1.00-1.12)	0.060	1658	88
Fever >=38.0 C	Multiple imputation (50 datasets), Firth	Body mass index, per kg/m²	1.06 (0.99-1.14)	0.072	1658	88

Outcome	Method	Covariate	Adjusted OR (95% CI)	p value	N	Events
Fever >=38.0 C	Multiple imputation (50 datasets), Firth	Nulliparity	5.84 (2.34-14.57)	<0.001	1658	88
Fever >=38.0 C	Multiple imputation (50 datasets), Firth	Cervical dilation, per cm	0.88 (0.64-1.22)	0.453	1658	88
Fever >=38.0 C	Multiple imputation (50 datasets), Firth	Ruptured membranes	0.95 (0.60-1.52)	0.841	1658	88
Fever >=38.0 C	Multiple imputation (50 datasets), Firth	Gestational diabetes	1.27 (0.76-2.11)	0.360	1658	88
Fever >=38.0 C	Multiple imputation (50 datasets), Firth	Hypertensive disorder	1.60 (0.72-3.57)	0.253	1658	88
Fever >=38.0 C	Multiple imputation (50 datasets), Firth	Dural puncture epidural	0.70 (0.44-1.09)	0.111	1658	88
Fever >=38.0 C	Multiple imputation (50 datasets), Firth	Analgesia duration, per 2 h	1.08 (1.00-1.17)	0.050	1658	88
Fever >=38.0 C	Multiple imputation (50 datasets), Firth	Pre-analgesia WBC, per 1 × 10^9/L	0.98 (0.88-1.08)	0.622	1658	88
Fever >=38.0 C	Multiple imputation (50 datasets), Firth	Calendar time, per month	0.88 (0.81-0.97)	0.007	1658	88
Fever >=38.0 C	Multiple imputation (50 datasets) plus IPOW logistic	Maternal age, per yr	1.06 (1.00-1.13)	0.052	1658	88

Outcome	Method	Covariate	Adjusted OR (95% CI)	p value	N	Events
Fever >=38.0 C	Multiple imputation (50 datasets) plus IPOW logistic	Body mass index, per kg/m²	1.07 (1.00-1.14)	0.053	1658	88
Fever >=38.0 C	Multiple imputation (50 datasets) plus IPOW logistic	Nulliparity	6.45 (2.52-16.46)	<0.001	1658	88
Fever >=38.0 C	Multiple imputation (50 datasets) plus IPOW logistic	Cervical dilation, per cm	0.91 (0.66-1.26)	0.567	1658	88
Fever >=38.0 C	Multiple imputation (50 datasets) plus IPOW logistic	Ruptured membranes	0.83 (0.51-1.37)	0.474	1658	88
Fever >=38.0 C	Multiple imputation (50 datasets) plus IPOW logistic	Gestational diabetes	1.24 (0.72-2.14)	0.441	1658	88
Fever >=38.0 C	Multiple imputation (50 datasets) plus IPOW logistic	Hypertensive disorder	1.90 (0.85-4.25)	0.118	1658	88
Fever >=38.0 C	Multiple imputation (50 datasets) plus IPOW logistic	Dural puncture epidural	0.67 (0.41-1.08)	0.098	1658	88
Fever >=38.0 C	Multiple imputation (50 datasets) plus IPOW logistic	Analgesia duration, per 2 h	1.08 (1.01-1.16)	0.030	1658	88

Outcome	Method	Covariate	Adjusted OR (95% CI)	p value	N	Events
Fever >=38.0 C	Multiple imputation (50 datasets) plus IPOW logistic	Pre-analgesia WBC, per 1 × 10^9/L	0.93 (0.84-1.04)	0.207	1658	88
Fever >=38.0 C	Multiple imputation (50 datasets) plus IPOW logistic	Calendar time, per month	0.86 (0.79-0.94)	0.001	1658	88
Any temperature elevation	Complete-case maximum likelihood (primary)	Maternal age, per yr	1.08 (1.03-1.13)	<0.001	1139	193
Any temperature elevation	Complete-case maximum likelihood (primary)	Body mass index, per kg/m²	1.02 (0.97-1.08)	0.415	1139	193
Any temperature elevation	Complete-case maximum likelihood (primary)	Nulliparity	3.23 (1.86-5.63)	<0.001	1139	193
Any temperature elevation	Complete-case maximum likelihood (primary)	Cervical dilation, per cm	0.86 (0.69-1.07)	0.166	1139	193
Any temperature elevation	Complete-case maximum likelihood (primary)	Ruptured membranes	0.91 (0.64-1.29)	0.583	1139	193
Any temperature elevation	Complete-case maximum likelihood (primary)	Gestational diabetes	0.96 (0.65-1.44)	0.861	1139	193

Outcome	Method	Covariate	Adjusted OR (95% CI)	p value	N	Events
Any temperature elevation	Complete-case maximum likelihood (primary)	Hypertensive disorder	0.88 (0.42-1.86)	0.739	1139	193
Any temperature elevation	Complete-case maximum likelihood (primary)	Dural puncture epidural	0.88 (0.62-1.23)	0.445	1139	193
Any temperature elevation	Complete-case maximum likelihood (primary)	Analgesia duration, per 2 h	1.13 (1.06-1.20)	<0.001	1139	193
Any temperature elevation	Complete-case maximum likelihood (primary)	Pre-analgesia WBC, per 1 × 10^9/L	1.04 (0.97-1.11)	0.266	1139	193
Any temperature elevation	Complete-case maximum likelihood (primary)	Calendar time, per month	0.88 (0.82-0.95)	<0.001	1139	193
Any temperature elevation	Multiple imputation (50 datasets), maximum likelihood	Maternal age, per yr	1.09 (1.05-1.13)	<0.001	1658	287
Any temperature elevation	Multiple imputation (50 datasets), maximum likelihood	Body mass index, per kg/m^2	1.02 (0.98-1.07)	0.275	1658	287

Outcome	Method	Covariate	Adjusted OR (95% CI)	p value	N	Events
Any temperature elevation	Multiple imputation (50 datasets), maximum likelihood	Nulliparity	3.20 (2.06-4.97)	<0.001	1658	287
Any temperature elevation	Multiple imputation (50 datasets), maximum likelihood	Cervical dilation, per cm	0.91 (0.76-1.09)	0.298	1658	287
Any temperature elevation	Multiple imputation (50 datasets), maximum likelihood	Ruptured membranes	1.03 (0.77-1.37)	0.840	1658	287
Any temperature elevation	Multiple imputation (50 datasets), maximum likelihood	Gestational diabetes	0.87 (0.62-1.23)	0.440	1658	287
Any temperature elevation	Multiple imputation (50 datasets), maximum likelihood	Hypertensive disorder	1.07 (0.58-1.98)	0.832	1658	287
Any temperature elevation	Multiple imputation (50 datasets), maximum likelihood	Dural puncture epidural	1.05 (0.78-1.41)	0.741	1658	287

Outcome	Method	Covariate	Adjusted OR (95% CI)	p value	N	Events
Any temperature elevation	Multiple imputation (50 datasets), maximum likelihood	Analgesia duration, per 2 h	1.14 (1.09-1.20)	<0.001	1658	287
Any temperature elevation	Multiple imputation (50 datasets), maximum likelihood	Pre-analgesia WBC, per 1 × 10^9/L	1.02 (0.96-1.08)	0.464	1658	287
Any temperature elevation	Multiple imputation (50 datasets), maximum likelihood	Calendar time, per month	0.88 (0.83-0.93)	<0.001	1658	287
Any temperature elevation	Multiple imputation (50 datasets) plus IPOW logistic	Maternal age, per yr	1.09 (1.05-1.13)	<0.001	1658	287
Any temperature elevation	Multiple imputation (50 datasets) plus IPOW logistic	Body mass index, per kg/m²	1.02 (0.98-1.06)	0.404	1658	287
Any temperature elevation	Multiple imputation (50 datasets) plus IPOW logistic	Nulliparity	3.04 (1.96-4.73)	<0.001	1658	287
Any temperature elevation	Multiple imputation (50 datasets) plus IPOW logistic	Cervical dilation, per cm	0.89 (0.74-1.08)	0.238	1658	287

Outcome	Method	Covariate	Adjusted OR (95% CI)	p value	N	Events
Any temperature elevation	Multiple imputation (50 datasets) plus IPOW logistic	Ruptured membranes	1.00 (0.74-1.34)	0.977	1658	287
Any temperature elevation	Multiple imputation (50 datasets) plus IPOW logistic	Gestational diabetes	0.85 (0.59-1.22)	0.377	1658	287
Any temperature elevation	Multiple imputation (50 datasets) plus IPOW logistic	Hypertensive disorder	1.19 (0.64-2.19)	0.587	1658	287
Any temperature elevation	Multiple imputation (50 datasets) plus IPOW logistic	Dural puncture epidural	1.04 (0.77-1.41)	0.784	1658	287
Any temperature elevation	Multiple imputation (50 datasets) plus IPOW logistic	Analgesia duration, per 2 h	1.12 (1.07-1.18)	<0.001	1658	287
Any temperature elevation	Multiple imputation (50 datasets) plus IPOW logistic	Pre-analgesia WBC, per 1 × 10^9/L	1.02 (0.95-1.09)	0.603	1658	287
Any temperature elevation	Multiple imputation (50 datasets) plus IPOW logistic	Calendar time, per month	0.88 (0.83-0.93)	<0.001	1658	287

Firth estimation was used for complete-case and multiple-imputation-only strict-fever models. Combined multiple-imputation plus IPOW models used unpenalized survey-weighted logistic regression for both outcomes. Multiple imputation addresses missing covariates and IPOW addresses measured predictors of outcome observation. Complete-case Firth intervals are profile penalized-likelihood intervals; pooled multiple-imputation intervals are Wald intervals combined with Rubin's rules.

Supplementary Table 5. Natural-spline likelihood-ratio test for analgesia duration.

Outcome	Comparison	Likelihood-ratio chi-square	df	p value	N	Events
Any temperature elevation	3-df natural spline vs linear duration	12.33	2	0.002	1139	193

The spline analysis was post hoc; the nominal exploratory p value tests departure from linearity and does not establish temporal order or causation.

Supplementary Table 6. Availability of post-analgesia laboratory measurements by temperature group.

Variable	Temperature group	Available, n/N (%)
Post-analgesia WBC	Normal temperature	153/1371 (11.2%)
Post-analgesia WBC	Elevation <38.0 C	142/199 (71.4%)
Post-analgesia WBC	Fever >=38.0 C	80/88 (90.9%)
Post-analgesia neutrophils	Normal temperature	153/1371 (11.2%)
Post-analgesia neutrophils	Elevation <38.0 C	142/199 (71.4%)
Post-analgesia neutrophils	Fever >=38.0 C	80/88 (90.9%)
Post-analgesia CRP	Normal temperature	81/1371 (5.9%)
Post-analgesia CRP	Elevation <38.0 C	72/199 (36.2%)
Post-analgesia CRP	Fever >=38.0 C	44/88 (50.0%)
Change in WBC	Normal temperature	130/1371 (9.5%)
Change in WBC	Elevation <38.0 C	131/199 (65.8%)
Change in WBC	Fever >=38.0 C	74/88 (84.1%)
Change in neutrophils	Normal temperature	130/1371 (9.5%)
Change in neutrophils	Elevation <38.0 C	131/199 (65.8%)
Change in neutrophils	Fever >=38.0 C	74/88 (84.1%)
Change in CRP	Normal temperature	21/1371 (1.5%)
Change in CRP	Elevation <38.0 C	16/199 (8.0%)
Change in CRP	Fever >=38.0 C	9/88 (10.2%)

CRP, C-reactive protein; WBC, white blood cell. Availability reflects clinical testing, not scheduled protocol measurement.

Supplementary Table 7. Exploratory ordinal-category correlations with Benjamini-Hochberg adjustment.

Variable	N	Spearman rho	Unadjusted p value	Benjamini-Hochberg q value
Analgesia duration, h	1651	0.219	<0.001	<0.001
Pre-analgesia WBC	1517	0.003	0.917	0.917
Post-analgesia WBC	375	0.055	0.286	0.327
Change in WBC	335	0.163	0.003	0.004
Post-analgesia neutrophils	375	0.236	<0.001	<0.001
Change in neutrophils	335	0.288	<0.001	<0.001
Post-analgesia CRP	197	0.353	<0.001	<0.001
Change in CRP	46	0.297	0.045	0.060

Correlations used variable-specific available records and are exploratory. The Benjamini-Hochberg procedure adjusted eight comparisons.

Supplementary Table 8. Repeated-identifier and BMI-range sensitivity analyses.

Outcome	Analysis	Covariate	Adjusted OR (95% CI)	p value	N	Events
Fever >=38.0 C	Primary	Nulliparity	8.05 (2.51-40.76)	<0.001	1139	61
Fever >=38.0 C	Primary	Dural puncture epidural	0.60 (0.35-1.02)	0.060	1139	61
Fever >=38.0 C	Primary	Analgesia duration, per 2 h	1.10 (1.00-1.20)	0.061	1139	61
Fever >=38.0 C	Primary	Calendar time, per month	0.93 (0.83-1.03)	0.176	1139	61
Any temperature elevation	Primary	Nulliparity	3.23 (1.86-5.63)	<0.001	1139	193
Any temperature elevation	Primary	Dural puncture epidural	0.88 (0.62-1.23)	0.445	1139	193
Any temperature elevation	Primary	Analgesia duration, per 2 h	1.13 (1.06-1.20)	<0.001	1139	193
Any temperature elevation	Primary	Calendar time, per month	0.88 (0.82-0.95)	<0.001	1139	193
Fever >=38.0 C	Repeated identifiers excluded	Nulliparity	7.79 (2.43-39.50)	<0.001	1138	60
Fever >=38.0 C	Repeated identifiers excluded	Dural puncture epidural	0.62 (0.36-1.06)	0.080	1138	60
Fever >=38.0 C	Repeated identifiers excluded	Analgesia duration, per 2 h	1.10 (0.99-1.20)	0.063	1138	60
Fever >=38.0 C	Repeated identifiers excluded	Calendar time, per month	0.93 (0.83-1.04)	0.199	1138	60
Any temperature elevation	Repeated identifiers excluded	Nulliparity	3.20 (1.84-5.57)	<0.001	1138	192

Outcome	Analysis	Covariate	Adjusted OR (95% CI)	p value	N	Events
Any temperature elevation	Repeated identifiers excluded	Dural puncture epidural	0.89 (0.63-1.25)	0.487	1138	192
Any temperature elevation	Repeated identifiers excluded	Analgesia duration, per 2 h	1.13 (1.06-1.20)	<0.001	1138	192
Any temperature elevation	Repeated identifiers excluded	Calendar time, per month	0.88 (0.83-0.95)	<0.001	1138	192
Fever >=38.0 C	BMI restricted to 15-50 kg/m²	Nulliparity	7.98 (2.49-40.46)	<0.001	1134	61
Fever >=38.0 C	BMI restricted to 15-50 kg/m²	Dural puncture epidural	0.60 (0.35-1.02)	0.060	1134	61
Fever >=38.0 C	BMI restricted to 15-50 kg/m²	Analgesia duration, per 2 h	1.10 (1.00-1.20)	0.061	1134	61
Fever >=38.0 C	BMI restricted to 15-50 kg/m²	Calendar time, per month	0.93 (0.83-1.03)	0.176	1134	61
Any temperature elevation	BMI restricted to 15-50 kg/m²	Nulliparity	3.19 (1.83-5.56)	<0.001	1134	193
Any temperature elevation	BMI restricted to 15-50 kg/m²	Dural puncture epidural	0.87 (0.62-1.23)	0.435	1134	193
Any temperature elevation	BMI restricted to 15-50 kg/m²	Analgesia duration, per 2 h	1.13 (1.06-1.20)	<0.001	1134	193
Any temperature elevation	BMI restricted to 15-50 kg/m²	Calendar time, per month	0.88 (0.82-0.95)	<0.001	1134	193

BMI, body mass index; CI, confidence interval; OR, odds ratio. Three repeated hospital identifiers occurred across six source rows, but only one linked complete-case record was excluded: n decreased from 1,139 to 1,138 and strict-fever events from 61 to 60.

Supplementary Table 9A. Questionnaire-linkage ambiguity sensitivity: Fever >=38.0 C.

Covariate	Primary OR (95% CI)	Ambiguous links excluded: OR (95% CI)	Exclusion p value
Maternal age, per yr	1.05 (0.98-1.13)	1.05 (0.98-1.13)	0.190
BMI, per kg/m²	1.04 (0.95-1.14)	1.04 (0.95-1.14)	0.373
Nulliparity	8.05 (2.51-40.76)	8.02 (2.51-40.62)	<0.001
Cervical dilation, per cm	0.90 (0.60-1.26)	0.90 (0.60-1.26)	0.580
Membrane rupture	0.89 (0.49-1.56)	0.88 (0.49-1.54)	0.665
Gestational diabetes	1.41 (0.76-2.50)	1.40 (0.76-2.49)	0.273
Hypertensive disorder	0.81 (0.21-2.26)	0.82 (0.21-2.28)	0.727
Dural puncture epidural	0.60 (0.35-1.02)	0.60 (0.35-1.03)	0.064
Analgesia duration, per 2 h	1.10 (1.00-1.20)	1.10 (1.00-1.21)	0.056
Pre-analgesia WBC, per 1 × 10^9/L	1.00 (0.89-1.11)	1.00 (0.89-1.11)	0.980
Calendar time, per month	0.93 (0.83-1.03)	0.93 (0.83-1.03)	0.166

Primary: n=1139, events=61; after excluding all flagged ambiguous links: n=1131, events=61. Firth penalized logistic regression; profile penalized-likelihood CI. Both models include all 11 listed covariates. This sensitivity analysis does not validate the remaining links or remove unmeasured linkage error.

Supplementary Table 9B. Questionnaire-linkage ambiguity sensitivity: Any questionnaire-reported temperature elevation.

Covariate	Primary OR (95% CI)	Ambiguous links excluded: OR (95% CI)	Exclusion p value
Maternal age, per yr	1.08 (1.03-1.13)	1.08 (1.03-1.13)	<0.001
BMI, per kg/m²	1.02 (0.97-1.08)	1.02 (0.97-1.08)	0.436
Nulliparity	3.23 (1.86-5.63)	3.24 (1.86-5.63)	<0.001
Cervical dilation, per cm	0.86 (0.69-1.07)	0.86 (0.69-1.07)	0.165
Membrane rupture	0.91 (0.64-1.29)	0.89 (0.63-1.27)	0.528
Gestational diabetes	0.96 (0.65-1.44)	0.96 (0.64-1.44)	0.851
Hypertensive disorder	0.88 (0.42-1.86)	0.89 (0.42-1.89)	0.768
Dural puncture epidural	0.88 (0.62-1.23)	0.88 (0.63-1.24)	0.473
Analgesia duration, per 2 h	1.13 (1.06-1.20)	1.13 (1.07-1.20)	<0.001
Pre-analgesia WBC, per 1 × 10^9/L	1.04 (0.97-1.11)	1.04 (0.97-1.10)	0.274
Calendar time, per month	0.88 (0.82-0.95)	0.88 (0.82-0.94)	<0.001

Primary: n=1139, events=193; after excluding all flagged ambiguous links: n=1131, events=193. Maximum-likelihood logistic regression; Wald CI. Both models include all 11 listed covariates. This sensitivity analysis does not validate the remaining links or remove unmeasured linkage error.

Supplementary Figure 1. Absolute standardized mean differences between the source registry and linked outcome records before and after weighting. The dashed reference is 0.10.

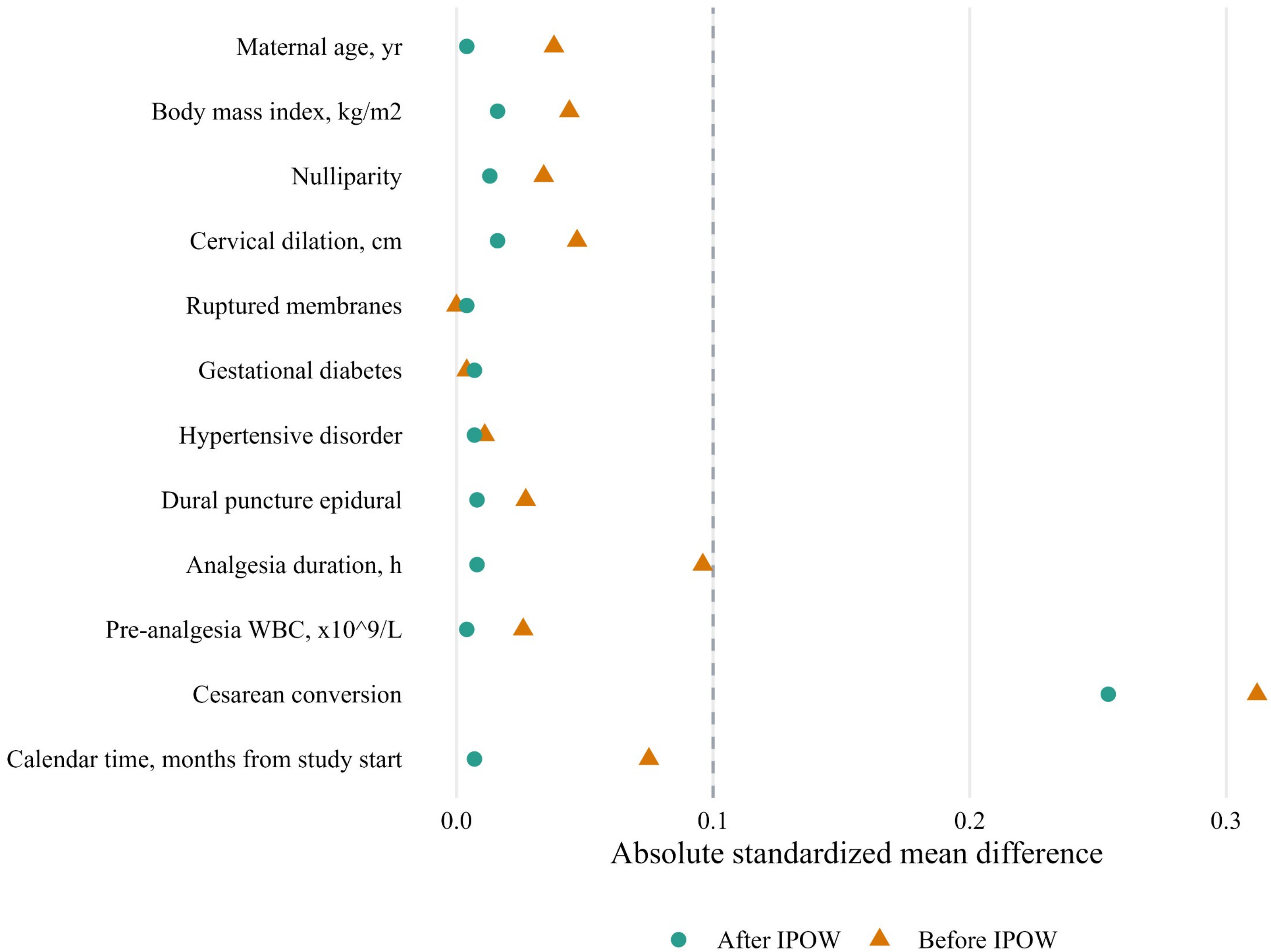

Supplementary Figure 2. Distribution of fitted questionnaire-linkage probabilities and truncated stabilized observation weights among linked records.

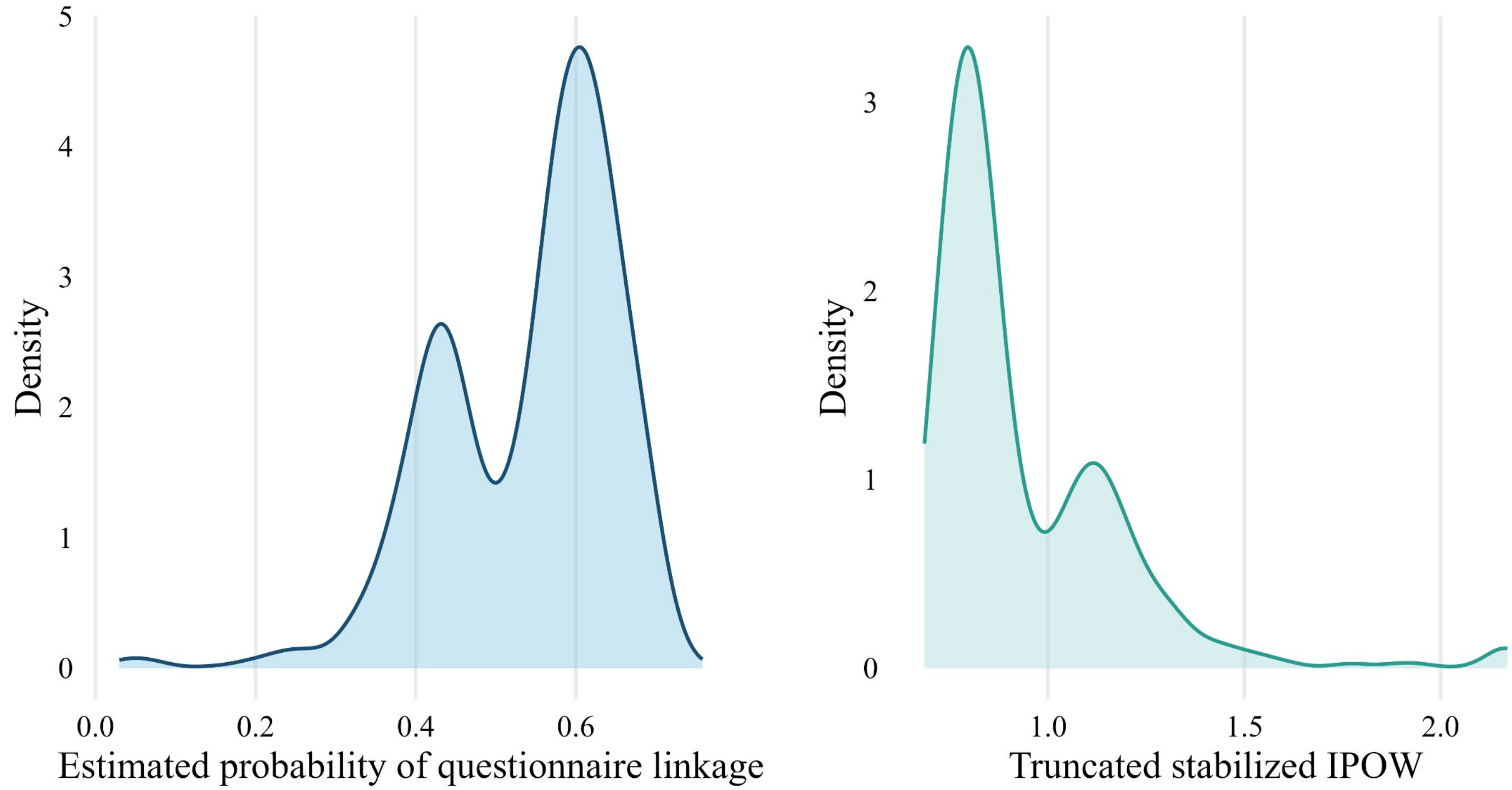

Supplementary Figure 3. Availability of post-analgesia laboratory measurements by questionnaire-derived temperature group. The figure displays the measurement process rather than a biological response.

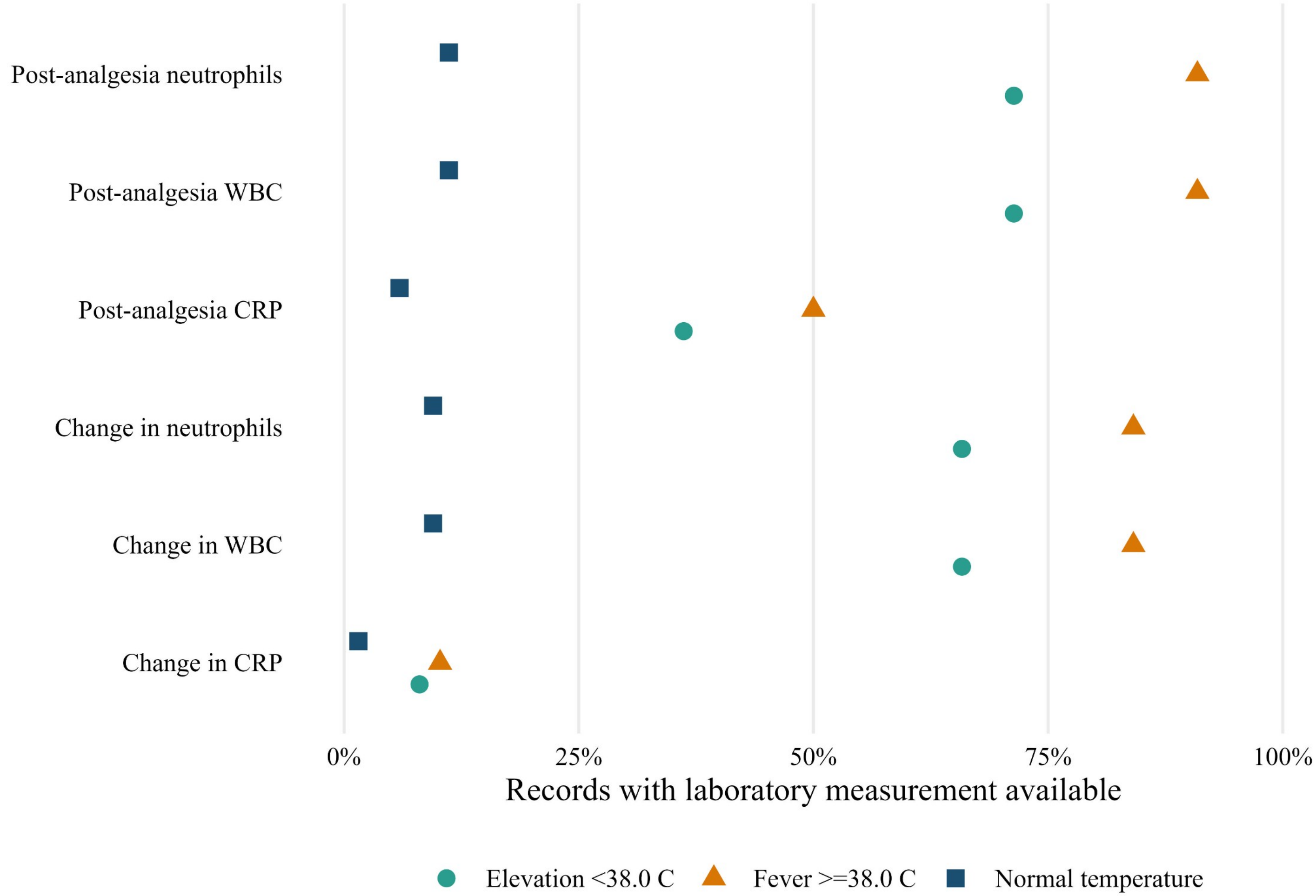

Supplement: Supplementary file 1 [file Datasheet1.pdf]
